# Acute kidney injury in the ICU: from injury to recovery: reports from the 5th Paris International Conference

**DOI:** 10.1186/s13613-017-0260-y

**Published:** 2017-05-04

**Authors:** Rinaldo Bellomo, Claudio Ronco, Ravindra L. Mehta, Pierre Asfar, Julie Boisramé-Helms, Michael Darmon, Jean-Luc Diehl, Jacques Duranteau, Eric A. J. Hoste, Joannes-Boyau Olivier, Matthieu Legrand, Nicolas Lerolle, Manu L. N. G. Malbrain, Johan Mårtensson, Heleen M. Oudemans-van Straaten, Jean-Jacques Parienti, Didier Payen, Sophie Perinel, Esther Peters, Peter Pickkers, Eric Rondeau, Miet Schetz, Christophe Vinsonneau, Julia Wendon, Ling Zhang, Pierre-François Laterre

**Affiliations:** 1 Australian and New Zealand Intensive Care Research Centre (ANZIC-RC), Department of Epidemiology and Preventive Medicine, Monash University, Melbourne, VIC Australia; 2 Department of ICU, Austin Health, Heidelberg, Australia; 3 Department of Nephrology, Dialysis and Transplantation, International Renal Research Institute of Vicenza (IRRIV), Vicenza, Italy; 4 Vice Chair Clinical Research, Department of Medicine, University of California San Diego, La Jolla, CA USA; 5 Département de Réanimation Médicale et de Médecine Hyperbare, Centre Hospitalier Universitaire, Angers, France; 6 Laboratoire de Biologie Neurovasculaire et Mitochondriale Intégrée, CNRS UMR 6214 - INSERM U1083, Université Angers, PRES L’UNAM, Angers, France; 7 Service de Réanimation Médicale, Nouvel Hôpital Civil, Hôpitaux Universitaires de Strasbourg, Strasbourg, France; 8 EA 7293, Fédération de Médecine Translationnelle de Strasbourg (FMTS), Faculté de médecine, Université de Strasbourg, Strasbourg, France; 9 Medical-Surgical ICU, Saint-Etienne University Hospital and Jean Monnet University, Saint-Étienne, France; 10 Medical ICU, AP-HP, Georges Pompidou European Hospital, Paris, France; 11 INSERM UMR_S1140, Paris Descartes University and Sorbonne Paris Cité, Paris, France; 12 AP-HP, Service d’Anesthésie-Réanimation, Hôpitaux Universitaires Paris-Sud, Université Paris-Sud, Hôpital de Bicêtre, Le Kremlin-Bicêtre, France; 13 ICU, Ghent University Hospital, Ghent University, Ghent, Belgium; 14 Research Foundation-Flanders (FWO), Brussels, Belgium; 15 ICU, University Hospital of Bordeaux, Bordeaux, France; 16 Department of Anesthesiology and Critical Care and Burn Unit, Hôpitaux Universitaire St-Louis-Lariboisière, Assistance Publique-Hôpitaux de Paris (AP-HP), University of Paris, Paris, France; 17 Département de Réanimation Médicale et de Médecine Hyperbare, CHU, Angers, France; 18 Ziekenhuis Netwerk Antwerpen, ZNA, Stuivenberg, Belgium; 19 Department of Intensive Care, Austin Hospital, Melbourne, VIC Australia; 20 Section of Anaesthesia and Intensive Care Medicine, Department of Physiology and Pharmacology, Karolinska Institutet, Stockholm, Sweden; 21 Department of Intensive Care, VU Medical Centre, Amsterdam, The Netherlands; 22 Department of Infectious Diseases, University Hospital, Caen, France; 23 Department of Biostatistic and Clinical Research, University Hospital, Caen, France; 24 Department of Anesthesia and Critical Care, SAMU, Lariboisière University Hospital, Paris, France; 25 Medical-Surgical ICU, Saint-Etienne University Hospital, Jean Monnet University Saint-Etienne, Saint-Étienne, France; 26 Department of Pharmacology and Toxicology, Radboud university Medical Center, Nijmegen, The Netherlands; 27 Department of Intensive Care Medicine, Radboud University Medical Center, Nijmegen, The Netherlands; 28 Urgences néphrologiques et Transplantation rénale, Hôpital Tenon, Université Paris 6, Paris, France; 29 Clinical Division and Laboratory of Intensive Care Medicine, Department of Cellular and Molecular Medicine, KU Leuven, Louvain, Belgium; 30 Service de Réanimation et Surveillance continue, Centre Hospitalier de BETHUNE, Bethune, France; 31 Kings College Hospital Foundation Trust, London, UK; 32 Department of Nephrology, West China Hospital of Sichuan University, Sichuan, Chengdu, China; 33 Intensive Care Unit, Saint Luc University Hospital, UCL, Louvain, Belgium

**Keywords:** Acute renal failure, Epidemiology, Renal replacement, Therapy, Anticoagulation, Renal blood flow, Extracorporeal epuration, Biomarkers

## Abstract

The French Intensive Care Society organized its yearly Paris International Conference in intensive care on June 18–19, 2015. The main purpose of this meeting is to gather the best experts in the field in order to provide the highest quality update on a chosen topic. In 2015, the selected theme was: “Acute Renal Failure in the ICU: from injury to recovery.” The conference program covered multiple aspects of renal failure, including epidemiology, diagnosis, treatment and kidney support system, prognosis and recovery together with acute renal failure in specific settings. The present report provides a summary of every presentation including the key message and references and is structured in eight sections: (a) diagnosis and evaluation, (b) old and new diagnosis tools, (c) old and new treatments, (d) renal replacement therapy and management, (e) acute renal failure witness of other conditions, (f) prognosis and recovery, (g) extracorporeal epuration beyond the kidney, (h) the use of biomarkers in clinical practice http://www.srlf.org/5th-paris-international-conference-jeudi-18-et-vendredi-19-juin-2015/.

## Definitions and classifications

Progress in disease management requires a systematic measurement of the underlying components, its natural history and influence on outcomes. The extent to which a disease can be identified and classified influences its recognition as a distinct entity, e.g., diabetes or myocardial infraction versus a syndrome, e.g., sepsis or vasculitis. Until recently, acute renal failure was considered a syndrome classified in a simplistic framework of pre-renal, renal and post-renal conditions attributed to multiple factors [1]. The absence of a standardized definition resulted in significant variation in reporting of this disorder and contributed to a lack of comparative data. Over the last 15 years, the syndrome has been renamed as acute kidney injury (AKI) and standardized diagnostic and staging criteria anchored to changes in serum creatinine (SCr) and urine output (UO) to define AKI [2]. The RIFLE/AKIN and Kidney Disease: Improving Global Outcomes (KDIGO) classification systems are based on identifying a minimal change in renal functional parameters that are related to an outcome and a gradation of severity that associates with incremental risk of worse outcomes [3]. Based on these principles, the current diagnostic and staging criteria have been widely accepted and tested for validity in several settings (Table 1), and they have been shown to perform well in being associated with adverse outcomes [4, 5]. However, our current criteria are still lacking in several respects and require further considerations and enhancements [3]. While an increase in creatinine is the hallmark of current criteria, several patients present to the hospital or clinic with an elevated creatinine with no prior values available. The absence of a “baseline” creatinine makes it difficult to establish a reference point to determine whether a rise has occurred and also to determine whether the patient recovers. Several different approaches have been suggested to compensate for a missing baseline value including estimation of a glomerular filtration rate (GFR) based on population norms [6], use of the nadir creatinine during hospitalization as baseline; however, these have all been found to result in over- or underestimation of AKI [7]. We have proposed differentiating the “baseline” creatinine from the “reference” creatinine. The former value is used to define a patients underlying kidney health status and should be based on the lowest value form >90 days prior to the AKI event [8]. The reference creatinine is the value used to determine the diagnosis of AKI and should be within 90 days of the event and can be the lowest value in that time period closest to the event that is being identified. This approach allows patients to be classified as having de novo AKI, AKI on chronic kidney disease (CKD) or AKI with unknown prior kidney health status. Transient increases in creatinine values are associated with better prognosis than persistent changes (>48 h); however, their risk of mortality is higher than those without any change in creatinine [4, 9]. The current definitions also do not include a decrease in creatinine as a criteria for AKI. Patients with an elevated creatinine that subsequently declines have been considered as community-acquired versus developing AKI during the hospital stay (hospital-acquired) and have a better prognosis [10]. In critically ill patients, factors influencing creatinine measurements including volume of distribution are often overlooked, leading to an under appreciation of the degree of renal dysfunction and delays in management [11]. Changes in UO have now been validated in several studies as early and sensitive criteria for AKI [12, 13]. However, in practice systematic measurement and recording of UO have been difficult and often urinary catheters are not placed given the risk of infection. The availability of several biomarkers of kidney injury has created excitement in offering new tools for recognition and management of AKI. Several biomarkers have been shown to have predictive ability in recognizing kidney damage earlier than creatinine but have not entered mainstream use as yet [14]. We have proposed considering biomarkers as functional (e.g., serum creatinine, serum cystatin C, UO) and damage markers (e.g., Kim-1, NGAL, TIMP2 and IGFBP3) and measure them in combination to improve the diagnostic categorization and permit more guided interventions [15]. These approaches will allow determination of a biomarker-positive creatinine negative stage as a measure of subclinical AKI. It is evident that while we have made significant advances in defining and staging AKI, there is much that is need to be done. We have the tools, knowledge and drive to continue to explore these areas with the goal to improve the lives of our patients.

**Table 1:**
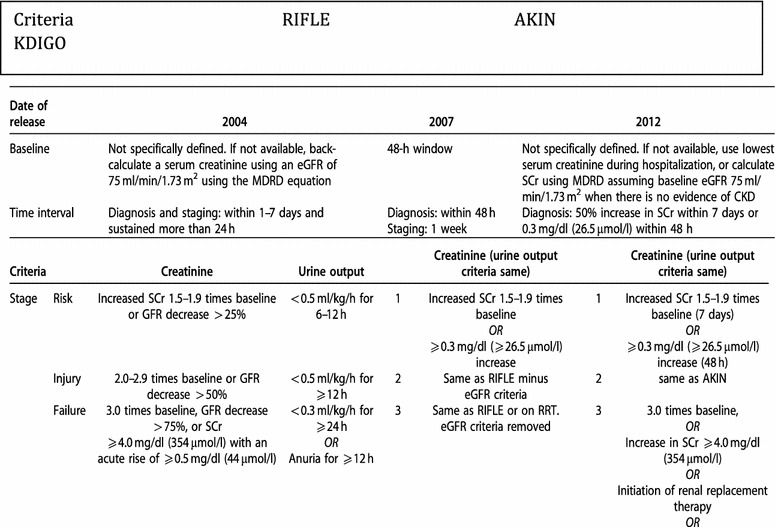
AKI scoring

See Ref. [234]

## RFR in normal and diseased kidney

Kidney function has been evaluated on the basis of GFR. Although average values of GFR have been identified for healthy subjects, there is no such a concept of normal GFR in the single individual. GFR represents a single-point assessment of kidney function that may be influenced by several factors and may not be a reliable marker of true filtration capacity since it remains in normal ranges until 50% of nephrons are lost [16, 17].

**Fig. 1:**
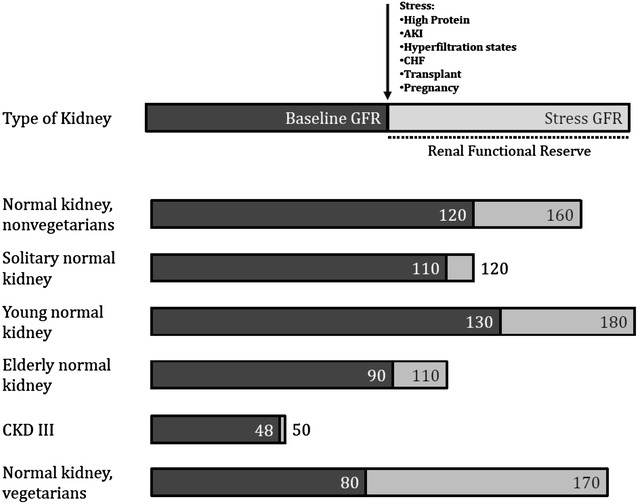
RFR and baseline GFR according to patients characteristics

Renal functional reserve (RFR) represents the kidney capacity to increase GFR in response to physiological or pathological stimuli. RFR can be clinically assessed by oral protein load or intravenous amino acid infusion and is defined as the difference between peak “stress” GFR after oral or i.v. protein load and the baseline GFR [18]. RFR and baseline GFR can be significantly different in subjects with different characteristics (Fig. 1). For patients with renal mass less than 50%, baseline and max GFR are often the same, unless a very low protein diet is in place [19].

RFR may be a reliable marker of the extent of “recruitable” GFR under renal stress. Thus, its reduction could be the earliest sign of both kidney frailty among healthy individuals and kidney damage after a single kidney injury.

RFR could be taken into account to establish a new stage of CKD. This stage may be named “stage 0” in case of a diminished RFR in the presence of a normal baseline GFR. The rationale for adopting RFR as the clinical parameter to diagnose “stage 0” CKD relies precisely on the clinical evidence that RFR reduction is the earliest subclinical sign of kidney function decline. This could be a situation, resulting in development of subsequent overt CKD and long-term complications. In many clinical scenarios, RFR reduction has been reported to be associated with reduced kidney function, and disease progression, earlier than GFR decline: RFR declines along with the progression of CKD, among pregnant women, those who present mild-to-moderate hypertension have lower RFR compared to normotensive pregnant women, hypertensive obese patients present a low RFR, in normotensive patients with systemic sclerosis with normal renal function and no urinary abnormalities, an abnormal RFR has reported to be associated with a greater than 5-year reduction in creatinine clearance, microalbuminuria and development of systemic hypertension, among male kidney donors, four weeks after nephrectomy, the GFR of the remaining kidney increase significantly, but GFR fails to raise after a protein load, demonstrating that although GFR is normal RFR has been lost.

As in CKD, the main topics on AKI perspectives and future directions include the prevention, earliest possible diagnosis and accurate prognosis estimation [2]. As the RFR has not been validated during the curse of AKI, it cannot be used for its diagnosis; moreover, it has not been evaluated the time extent that it takes for RFR to return to its “best possible” levels. At this point, it has not been established if renal function returns to its best possible baseline immediately after the AKI trigger has stopped, or if it exists a period of time in which dysfunctional, but yet viable nephrons remains dysfunctional, thus being possible to create a concept such as “stunned nephrons” the same way stunned myocardium represents a well-recognized entity [20].

Moreover, RFR measurement is also valid in critical care scenarios. RFR may be useful on the evaluation of response to diuretics (i.e., patients with a lower RFR most likely will not respond to diuretic) the same way alveolar recruitability measurements indicate the extent in which high PEEP may actually improve oxygenation among acute respiratory distress syndrome (ARDS) patients, and to evaluate the initiation of renal replacement therapy (RRT), since patients with a low RFR much more probably will require RRT [21].

Taking this into account, it is reasonable to add a new “susceptibility stage” also in the evaluation of AKI, since identifying an stage 1 AKI does not allow to identify and stratify the risk of AKI on the base of objective, single-value criteria. Also, an “susceptibility stage” AKI may be used to indicate the patients that, after AKI, have lost its baseline RFR and thus remains susceptible to a new AKI episode, even in the absence of elevation of SCr or tubular damage biomarkers levels [22].

In conclusion, RFR is an interesting concept, and it represents an objective and dynamic measurement of renal function that may be useful on the early detection of kidney susceptibility for either acute or chronic kidney injury. It may also be used to stratify renal risk, evaluate the best treatment maneuver, and measure both functional renal recovery after AKI and renal disease progression in the case of CKD. It remains unclear whether or not its measurement under critical conditions may be valid, whether RFR value may be used to predict response to specific therapeutic maneuvers susceptibility to nephrotoxic drugs, and the extent of its value to prognosticate long-term renal loss after a single AKI. In this context, more prospective clinical trials on the evaluation of the forenamed applications for RFR [23].

## Epidemiology of AKI in the ICU: Are there any changes?

Over the last decade, AKI has come to prominence as a major contributor influencing outcomes in critically ill patients. With the development of the RIFLE/AKIN/KDIGO diagnostic systems [3], several reports have described the epidemiology of AKI in the ICU. These have ranged from descriptions of administrative data sets, retrospective analysis of single- and multicenter cohorts and prospective cohort studies [24–27]. It has been difficult to compare the data across centers; however, some common themes have emerged. The incidence of AKI is now believed to be significantly higher than previously believed with over 50% of patients in the ICU developing stage 1 AKI at some point during the course, while stages 2 and 3 AKI are considerably less and RRT requirement is approximately 10% (Table 2). The staging system has been demonstrated to be a good predictor of outcomes with an increasing risk of mortality and resource utilization with higher stages regardless of the setting. Risk factors have included increasing age, presence of heart failure, liver failure and CKD and anemia and exposures to nephrotoxic agents including antibiotics, NSAIDS and contrast. Infections, sepsis, shock, need for mechanical ventilation and surgery are well recognized as high-risk settings for the development of AKI [28]. There is increasing recognition that patients may present to the ICU with AKI (community-acquired) or develop it during the hospital stay (hospital-acquired). The latter is associated with a worse prognosis and is often iatrogenic in nature [10]. Management strategies continue to reflect supportive measures focusing on fluid delivery, diuretics, avoidance of nephrotoxic agents and RRT for the most severe cases; however, there have not been any specific measures targeted to the kidney that have been successful [29]. There is increasing recognition that fluid accumulation and overload contribute to adverse outcomes although it is uncertain whether this is causal. Renal recovery from ICU AKI has been variably reported as there are no standard definitions in this regard. There is a growing concern that AKI contributes to a significant burden of CKD, and long-term follow-up studies report poor renal outcomes. Two large prospective multicenter international studies provide additional evidence of the heterogeneity of AKI in ICU patients and report significant differences in risk factors etiology and management and outcomes based on available resources. Bouchard et al. [25] have shown that patients in emerging countries were more likely to have glomerulonephritis (GN) and acute interstitial nephritis, while those in developed countries had higher reported rates of pre-renal AKI, sepsis and acute tubular necrosis. Residence in an emerging country was associated with more than a twofold increase in hospital mortality and a threefold lower rate of renal recovery in survivors. Hoste et al. [24] found similar results with a significant relationship to the underlying gross national income. Based on the accumulated evidence so far, it is evident that AKI continues to be major problem for critically ill patients worldwide [30]. Identification of high-risk patients coupled with early diagnosis facilitated by emerging biomarkers and surveillance through electronic medical records are being proposed as opportunities to improve outcomes [31]. Strategies to prevent AKI and its consequences with targeted interventions are sorely needed; however, it will require continued multidisciplinary team efforts to optimize and standardize AKI management to make a difference in this devastating complication.

**Table 2:** Incidence of AKI in critically ill patients

	Year	N ICU	# Patients	RIFLE/AKIN/KDIGO	Creat/UO	Incidence (%)
Hoste	2006	7	5383	RIFLE	Creat and UO	67
Ostermann [12]	2007	22	41,972	RIFLE	Creat	35.8
Ostermann [13]	2008	22	22,303	AKIN	Creat	35.4
Bagshaw [14]	2008	57	120,123	RIFLE/AKIN	Creat and UO	37.1
Joannidis [15]	2009	303	16,784	RIFLE/AKIN	Creat and UO	35.5
Mandelbaum [16]	2011	7	14,524	AKIN	Creat and UO	57
Nisula [17]	2013	17	2091	AKIN	Creat and UO	39.3
Liborio [18]	2014	1	18,410	KDIGO	Creat and UO	55.6
Kellum [19]	2014	8	32,045	KDIGO	Creat and UO	74.5
Hoste [2]	2015	97	1802 (1032)*	KDIGO	Creat and UO	57.3
Bouchard [9]	2015	9	6647 (745)*	AKIN	Creat	19.2

Screened (AKI)

## Place of renal biopsy in the ICU

AKI results from several systemic aggressors such as sepsis, shock, nephrotoxic drugs and major surgery. Indeed, these aggressors were observed in a vast majority of ICU patients with SCr elevation analyzed in the BEST study [26]. An article described a series of 19 consecutive patients who died of septic shock and were systematically biopsied immediately after death [32]. The renal lesions on pathological showed various degrees of acute tubular injury, vascular leukocytic infiltration, fibrin deposition and apoptosis. Another study reported similar lesions [33]. Thus in the setting of AKI factors, a uniform pattern of renal lesions (most often referred as acute tubular necrosis even though this term is an oversimplification) is reproducibly observed. As no modification of treatment can be derived from this pattern, renal sampling cannot be advocated in such patients in clinical routine.

However, using AKI staging criteria in the ICU setting should not lead to the assumption that all patients with acute SCr elevation have actually AKI. Indeed, some patients may suffer from a more specific «nephrologic» form of acute renal failure whose prompt diagnosis and treatment are crucial. In the BEST cohort of critically ill patients with SCr increase, 12% of the patients had “other” factors than usual AKI factors identified [26]. Reviewing all renal biopsies performed in a nephrology department for acute renal failure, Uezono et al. observed among patients aged 65 years and older, 71% had a final diagnosis of crescentic GN [34]. In a series of 49 biopsies in patients with renal failure and acute infectious endocarditis, the most common biopsy finding was necrotizing and crescentic GN (53%), followed by endocapillary proliferative GN (37%) [35].

Two recent studies described the diagnostic yield of renal biopsy in ICU patients in whom the diagnosis of AKI was doubtful [36, 37]. These retrospective studies were performed in France on a 10-year period on 15 ICUs. They retrieved “only” 133 biopsies (native kidneys in 124), indicating that this procedure was performed rarely (more than 100,000 patients having being admitted in these ICU during the study period). In Augusto study, in nearly 90% of cases, biopsy was performed percutaneously under ultrasonographic guidance, a few patients having CT-scan-guided or surgical biopsy. The rate of adverse events in the two studies ranged from 12 to 22%, and the rate of serious events (shock or requirement for >2 red cell packs) was being similar at 12% with one death overall. This high frequency of serious adverse events is tenfold higher than observed in the nephrology setting. In one of the studies, the rate of adverse event was significantly increased when the platelet count was below 200 G/L [36]. Transjugular biopsy may represent an interesting alternative to percutaneous sampling in high-risk patients even though no study with this technique has been dedicated to ICU patients. On native kidneys, the two studies showed a similar diagnostic yield, with around half of the patients having a specific diagnosis other than acute tubular necrosis. These diagnoses give a very interesting insight into what should be considered in ICU patients with acute elevation in SCr beyond AKI. The most frequent diagnoses were crescentic glomerulopathy with vasculitis (most patients having a final diagnosis of ANCA-associated vasculitis), thrombotic microangiopathy and acute GN (associated with endocarditis in most cases). Interestingly, a few patients had end-stage renal lesions on the renal biopsy, showing that they had been initially misdiagnosed as acute renal failure. The result of the biopsy was judged as having an impact on treatment in between 41 and 71% of the cases (reflecting variation in how an impact was defined). Notwithstanding, in these two studies whether the final diagnoses could have been established using alternate approaches such as serum antibodies screening panel or biopsies at other sites than the kidney was not disclosed. These alternate approaches may be of high value. For example, in a study, a pre-biopsy clinical diagnosis of ANCA-associated GN was 100% correct showing the usefulness of ANCA testing [34].

In one of these studies, some factors were observed associated with a greater likelihood of having a pathological diagnosis other than acute tubular necrosis: any extrarenal sign that evokes a systemic disease (i.e., arthritis), absence of any usual AKI factor before creatinine rise, occurrence of renal creatinine increase before hospital admission and any abnormal result on autoimmune/microangiopathic screening [36]. These factors may be helpful to identify patients in which particular attention should be paid to the cause of renal dysfunction. Renal biopsy may be then considered if a thorough noninvasive approach had failed, and weighing the high risk of hemorrhagic adverse events.

## Evaluation of renal blood flow by renal Doppler

Despite our increasing ability to support vital organs and resuscitate patients, the morbidity and mortality of AKI remain high in the ICU. The ability to predict the occurrence of AKI is crucial for the development of preventive strategies. Early diagnosis of AKI requires markers that are sensitive and easily applicable in clinical practice. The use of Doppler ultrasonography to assess renal perfusion is increasing in many kidney diseases and in the ICU. The Doppler-based renal-resistive index, which is a simple, rapid, noninvasive and repeatable marker, could be a promising tool to detect early patients, which are the most at risk of developing AKI in ICU and to distinguish transient from persistent AKI. Moreover, the resistive index could also be useful to guide therapeutic strategies to improve kidney perfusion at the bedside. The recent progress in ultrasound with contrast-enhanced ultrasound (CEUS) gives the opportunity to assess not only the kidney macrocirculation but also the kidney microcirculation in the ICU. CEUS could be a precise and reproducible way to evaluate renal perfusion in ICU. Further studies are required to validate CEUS in ICU and to establish whether there is a correlation between changes in CEUS-derived indices and markers of renal function and outcome. CEUS is currently a research tool, but perhaps in the future CEUS could assess the renal microcirculation at the bedside in the usual clinical practice.

## Old and new diagnostic tools: how to use these in clinical practice

### Introduction

**Fig. 2:**
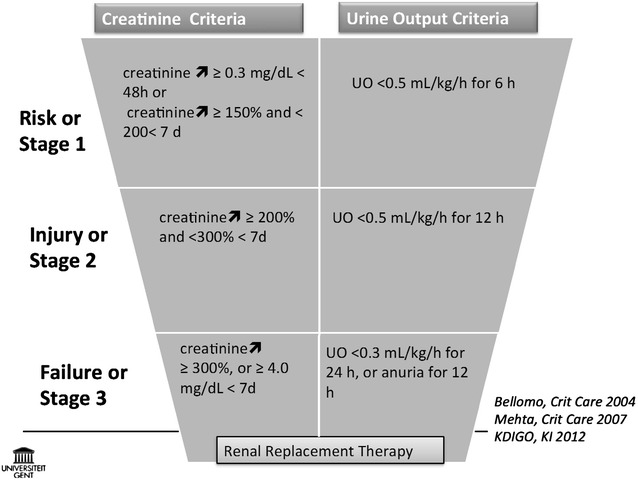
KIDGO definition for AKI

Kidney function is in ICU patients traditionally evaluated by SCr and UO. These parameters are also used in the current KDIGO definition for AK (Fig. 2) [38].

### Urine output

UO is probably the most readily available parameter for assessment of kidney function. The KDIGO classification requires hourly measurement of UO. Since ICU patients generally have a urinary bladder catheter, this requirement is easily met.

Unfortunately, several extrarenal factors can lead to false-positive or false-negative readings of kidney function. For instance, kinking of the urinary catheter may falsely indicate oliguria, while use of diuretics can give a false impression of good kidney function. Also, varying time intervals between UO recordings may hinder correct interpretation of the KDIGO criteria.

### Serum creatinine

In non-ICU patients, SCr is predominantly determined by urinary clearance. However, in ICU patients altered production and volume of distribution of Cr will also affect its concentration. Cr is metabolized from creatine, which is released at a relative constant rate from muscles. However, bed rest and critical illness polymyoneuropathy will decrease muscle mass and so lower SCr. Also, volume resuscitation and increased volume of distribution will dilute SCr. Furthermore, changes in clearance will only be translated with a delay in SCr. As a consequence, single-point SCr may underestimate kidney function.

For these reasons, AKI is defined by a change in SCr (Fig. 2). This requires the knowledge of a baseline SCr. In case this is not available, an MDRD-derived baseline value is proposed.

### UO and SCr: the KDIGO criteria

AKI is staged on the worst of either SCr or UO criteria. This suggests that a patient classified on UO criteria has similar AKI severity and outcomes as when defined by SCr. However, several studies have shown that UO criteria are more sensitive and associated with better outcomes; and when a patient meets both SCr and UO for a certain stage, outcomes are marked more worse [13].

### AKI sniffer or electronic alert for KDIGO stages

Several small and observational studies have shown that early intervention can improve outcomes. The use of electronic tools that alert when KDIGO criteria are met can so be of help. Wilson et al. could not show a difference in outcomes in a hospital-wide setting [39]. A finding that may be explained by the absence of changes in care follows the alert. We found in our ICU that a sniffer alert leads to more and earlier interventions and also a trend for less progression of AKI [40]. These conflicting findings may be explained by the ICU versus hospital-wide setting, but also by single-center design.

### Kidney function or GFR

In out-patients, creatinine clearance (CCr) or estimated GFR (eGFR) can be assessed by simple equations such as MDRD or CKD-EPI. Alterations in muscle mass and volume of distribution limit the validity of these in ICU patients, which explains why eGFR is not adequate in ICU patients [41].

Measured urinary Ccr (Urinary Cr × Urinary volume)/(Scr × time) over a 2 to 24-h time interval is therefore still the only reliable and simple way to assess kidney function in ICU patients.

### Cystatin C

Cystatin C is a small protein produced by nucleated cells and eliminated by GFR. It behaves therefore similar to SCr, but is less dependent on muscle mass. In ICU patients, cystatin C will detect AKI 1–2 days earlier before SCr. However, it performed worse to SCr in cardiac surgery patients. At present, the price (5–10 times that of SCr) also limits its daily use.

### Pre-renal AKI or transient AKI

There are several urinary indices for transient AKI. Most commonly used are urinary Na^+^, fractional excretion of Na^+^ (FENa) and FE of urea. Studies on their use showed conflicting results. Currently, we can therefore not recommend their use.

### AKI detection before GFR decrease: damage

Before actual decline of GFR with resulting changes in SCr and UO, the kidney is exposed to stress and damage. Several biomarkers can indicate this and so may help in early recognition of AKI.

In burn patients, proteinuria is strongly associated with the development of AKI. In expert hands, the so-called urine sediment score can also indicate the risk of AKI.

At present, we have also two new biomarkers at our disposal: NGAL and TIMP-2*IGFBP7. Many others such as KIM-1 and chitinase-3-like protein 1 (CHI3L1) are under evaluation. These biomarkers allow more early identification of AKI, but also provide us new insights into the pathogenesis of AKI. In addition, they may indicate use of RRT, renal recovery and long-term outcomes. Detailed info on their use will be discussed in the chapter on the pro–con debate on these biomarkers.

### Old and new drugs: diuretics

**Fig. 3:**
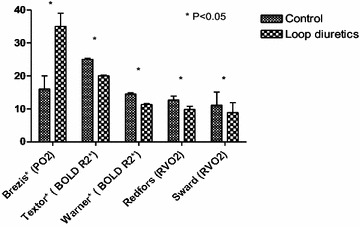
Changes in renal oxygen consumption following loop diuretics. In human or animal, use of diuretics is associated with higher renal medulla PO2 (mmHg), lower R2* BOLD MRI signal (Hz, 1/s) suggesting a higher oxygenation and lower renal oxygen consumption (RVO2, ml/min) in various conditions. *Results from selected studies (Brezis et al. Am J Physiol Renal Physiol 1994; Textor et al. J Am Soc Nephrol 2009; Warner et al. Invest Radiol 2011; Redfors et al. Intensive Care Med 2009; Sward et al. Intensive Care Med 2005). R2* is believed to be correlated with deoxyhemoglobin and therefore inversely correlated with tissue oxygenation

Physiological effects of diuretics might help in mitigating renal injury. Furosemide acts in inhibiting the active Na^+^/K^+^/Cl^−^ co-transport pump on the luminal cell membrane surface of the medullary thick ascending limb of Henle loop. Tubular sodium reabsorption is an expensive mechanism accounting for the larger part of the oxygen consumption in an outer medulla already exposed to ischemic damage. Both animal and human studies demonstrated diuretics to limit active sodium reabsorption ultimately decreasing both relative medulla hypoxia and oxygen consumption (Fig. 3) [42, 43]. Additionally, furosemide has been shown to attenuate apoptosis following ischemia–reperfusion injury in experimental model [44].

These theoretical benefits are, however, still to be validated in clinical setting. Thus, although widely used [27, 45], diuretics have failed to demonstrate any benefit in preventing AKI, limiting the risk of RRT or fastening renal recovery [46]. In a recent systematic review assessing the influence of diuretics in 876 patients, diuretics use was not associated with survival (relative risk 1.02; 95% CI 0.86–1.19) or with reduced need for RRT (RR 1.12; 95% CI 0.93–1.34) [46]. Cohort studies [27] and randomized trials [47] even suggested diuretics to be harmful in specific subgroups. Lack of adequately powered randomized controlled study (RCT) and variables unaccounted for such fluid balance changes or clustering effect are, however, to be taken into account when interpreting these negative results. Several advances in this field are to be expected.

First, as stated above, diuretics lack of efficacy has to be tempered down with regard to the high risk of bias in available studies. Large and adequately randomized control trials in AKI patients are currently ongoing [NCT01275729, NCT00978354; last accessed on June 20 2015] and should put an end to remaining uncertainties in field.

Additionally, several specific uses of diuretics are being evaluated. Loop diuretics gain access to tubular lumen through active secretion, and their action is therefore dependent of tubular function [48]. Response to loop diuretic stress test might reflect degree of tubular injury and has been assessed in way to evaluate short-term renal prognosis. In a preliminary study, Chawla and colleagues demonstrated furosemide stress test to be a potent predictor of progression to AKI stage III or need for RRT [48, 49]. Moreover, performance of diuretic test for these purposes was higher than that of most serum or urinary biomarkers [49]. Although encouraging, it must be noted that progressors toward AKI stage III had lower urinary output [48], were more likely to have oliguria before diuretic test and had more frequently an AKI stage II [49]. Performance of the furosemide stress test was not adjusted for these confounders, and additional studies are therefore needed to confirm these promising results. The RENALGUARD system has been developed in way to achieve high UO with diuretics while simultaneously maintaining fluid balance via real-time crystalloid compensation [50]. This system has been tested with interesting results in preventing contrast-associated nephropathy (CAN) [50]. Respective influence of this device-led “forced diuresis” and of changes in urinary creatinine excretion following diuretics use remains, however, to be delineated. This device might nevertheless be useful in specific niches requiring increased tubular flow and avoidance of dehydration to limit tubular injury such prevention of specific drug nephrotoxic effects or tumor lysis syndrome. Last, this device may provide opportunities for physiological research in allowing assessment of renoprotective effects of diuretics while ensuring neutral fluid balance. The last and most obvious potential interest of diuretics remains in limiting fluid overload. Increasing number of evidences pointed out the deleterious effects of positive fluid balance [51]. Not only recent studies underlined the poor outcome associated with positive fluid balance, but also they underlined the negative impact of positive fluid balance on various organs, including kidneys. Thus, renal congestion, interstitial edema and subsequent changes in renal perfusion are likely to participate in AKI development. In this regard, diuretics are first and above all already potent and validated drugs in allowing fluid balance adjustment.

Despite being widely used since half a century, uncertainties regarding potential interests of loop diuretics in AKI patients remain. The available evidences argue against routine use of diuretics at bedside in preventing or treating AKI. Physiological and preliminary studies, however, clearly underline potential renal benefits of loop diuretics. Whether these theoretical benefits may translate into clinically relevant benefits is yet to be proven.

### Optimizing arterial pressure in patients with septic shock to prevent acute renal failure in ICU?

During septic shock, optimizing arterial pressure to prevent acute renal failure remains a challenge for intensivists. The latest Surviving Sepsis Campaign guidelines [52] recommend (grade 1C: strong recommendation based on low level of evidence) that mean arterial pressure (MAP) should be targeted above 65 mmHg. However, there are few evidence-based data to support this threshold, as far as organ perfusion and dysfunction are concerned. The guidelines therefore temper this target by highlighting that “*optimal MAP should be individualized as it may be higher in patients with atherosclerosis and/or previous hypertension than in young patients without cardiovascular comorbidity.*” Shock resuscitation is a subtle balance between the risk of hypotension that would be responsible for organ hypoperfusion and subsequent dysfunction, and an excessive vasoconstriction associated with higher MAP target that requires increased vasopressors infusion rates, which would also result in organ hypoperfusion. Therefore, the way to prevent acute renal failure, while avoiding complications related to higher MAP and higher vasopressor need, would be to determine low and high MAP thresholds for resuscitation of septic shock patients.

### Is it possible to determine a low MAP threshold to prevent acute renal failure for resuscitation of shock patients with sepsis?

Several studies investigated the effects of a MAP level on acute renal failure. Thus, in a retrospective cohort study of 274 septic patients, Dünser et al. [53] showed that, if there was a linear association between the time when MAP was below 60 mmHg during the first 24 h after ICU admission and 28-day mortality, the need for RRT was highest when MAP was below 75 mmHg. The authors therefore suggested that a higher MAP could be necessary to maintain renal function. More recently, Legrand et al. [54] showed that diastolic arterial pressure during the first 24 h after ICU admission was significantly lower, along with a higher central venous pressure, in patients who would develop acute renal failure.

### Is it possible to determine a high MAP threshold to prevent complications related to a higher MAP and higher vasopressor needs for resuscitation of shock patients with sepsis?

Several prospective studies attempted to increase MAP by increasing norepinephrine infusion rates, but most of them included a small number of patients, with a short-term follow-up, and none reported beneficial effect on renal function. In a post hoc analysis of 290 patients of a multicenter trial in which MAP was maintained above 70 mmHg during shock, Dünser et al. [55] showed that a MAP ≥ 70 mm Hg was not associated with increased mortality, but elevating MAP above 70 mmHg by increasing vasopressor infusion rates was associated to the development of disease-related events and increased 28-day mortality. Poukkanen et al. [56] later prospectively confirmed in 423 patients with severe sepsis that vasopressor load was higher in patients with progression of acute renal failure.

In the SEPSISPAM trial [57], 778 patients with septic shock were stratified according to previous hypertension history and were treated with “*low*” (65–70 mmHg) versus “*high*” (80–85 mmHg) MAP target. In patients with previous hypertension treated with the high MAP target, there was significantly less renal failure—as defined by the doubling of plasma creatinine (38.8 versus 52.0%, *p* < 0.05)—and less requirement for RRT between day 1 to day 7 (31.7 versus 42.2%, *p* < 0.05). Conversely, for patients without prior hypertension, there was no benefit to increase MAP target. As a reminder, there was no difference for 28-day mortality, whatever the MAP group, and the occurrence of de novo atrial fibrillation was more frequent in the group treated with a higher MAP, most likely due to the higher vasopressor requirements.

The pathophysiological mechanisms of sepsis-induced acute renal failure are still a matter of debate. When arterial pressures are low, renal autoregulation adaptation is lost and renal vascular resistances are increased, with subsequent renal hypoperfusion and ischemia. However, acute renal failure may still occur during hyperdynamic sepsis despite increased total renal blood flow, suggesting that other mechanisms are involved. Renal cortical microcirculatory flow is also impaired from the early stages of sepsis, before the renal perfusion pressure (RPP) decreases. Several mechanisms are therefore likely to lead to sepsis-induced renal dysfunction, including hypoperfusion, venous congestion, microcirculation alterations, but also mechanisms independent of hemodynamic impairments, like inflammation and oxidative stress.

### Conclusion

Although the exact pathophysiological mechanisms, but also the weight of each mechanism, are still debated, increasing MAP during septic shock might therefore benefit to patients with previous hypertension and prevent acute renal failure. However, the increase in MAP is associated with increased vasopressor load, which in turn may increase adverse events and especially cardiac side effects.

### Alkaline phosphatase: serendipity and the discovery of its renal-protective properties

From a putative antisepsis agent to a renal-protective therapy currently investigated in a large phase II clinical trial. Alkaline phosphatase (AP) is a dephosphorylating enzyme naturally occurring in the human body. The enzyme is located in several organs, including the kidney, liver, intestines, bone and placenta, where it is involved in, for example, bone mineralization, regulating of intestinal barrier function and disease prediction. Next to its physiological role, AP plays a role in host defense and innate immunity. The anti-inflammatory role of AP was already demonstrated in the late nineties by Poelstra et al., who found that inhibition of endogenous AP in rats exposed to a sublethal dose of gram-negative *Escherichia coli* resulted in significant higher mortality rates. This observation was confirmed by several other in vivo studies. Exogenous placental and intestinal AP improved survival rates, reduced systemic peak cytokine and nitric oxide levels and prevented liver and lung damage during systemic inflammation in mice. In sheep, the administration of intestinal AP attenuated plasma interleukin-6 levels and improved gas exchange during fecal sepsis, whereas intestinal AP enhanced thrombocyte counts in endotoxemic piglets. These effects are all attributed to dephosphorylation and thereby detoxification of lipopolysaccharide (LPS), a key player in the pathogenesis of sepsis.

### Clinical trials with biAP

**Fig. 4:**
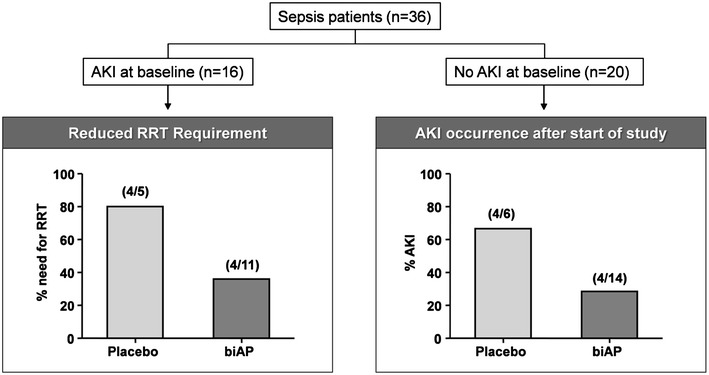
RRT requirement and AKI occurence after biAP in patient with sepsis

Considering the profound anti-inflammatory effect in the preclinical tests and its presumed mechanism of action, sepsis trials with AP in men were initiated. First, bovine-derived intestinal AP (biAP) was administered to healthy volunteers and severe sepsis patients to assess the pharmacokinetic properties and confirm safety [58]. Subsequently, a multicenter phase II clinical trial was conducted with 36 patients with severe sepsis or septic shock admitted to the ICU. Patients were randomized to receive biAP or placebo intravenously for 24 h. No statistically significant effects of biAP on plasma cytokine levels or other systemic inflammatory parameters were observed, but in a subpopulation of patients with AKI (protective effects appeared to be present [59]. Treatment with biAP significantly attenuated the increase in median plasma creatinine levels and the urinary excretion of a marker of proximal tubule injury, glutathione-S-transferase A1. Also, the need for and duration of RRT tended to be less, whereas patients without a diagnosis of AKI at inclusion were less likely to develop AKI (Fig. 4). Although these findings were not statistically significant, due to the small number of patients, based on these results the renal effects of biAP were further explored in a second phase II clinical trial. Again, 36 patients with severe sepsis or septic shock and evidence of early AKI were randomized to biAP or placebo intravenously for 48 h. Patients treated with biAP showed improved endogenous CCr and reduced the need for, and duration of RRT, confirming its renal-protective effects. In addition, biAP infusion attenuated the urinary excretion of renal injury markers interleukin-18 and kidney injury molecule-1 compared to placebo [60].

### Human recombinant alkaline phosphatase

As the protective effect of AP was demonstrated in a limited number of patients only, results needed to be reconfirmed in larger trials. However, administering bovine-sourced material to humans is less desirable due to the risk of immune reactions and challenges obtaining bovine spongiform encephalopathy (BSE)-free sources of AP. Therefore, a human AP was developed. By replacing the crown domain of a human intestinal AP with the crown domain of human placental AP, a recombinant AP (recAP) was obtained that is highly stable, biologically active and has beneficial pharmacokinetic properties compared to biAP. The protective effect of recAP was recently demonstrated during LPS-induced inflammation in a human renal cell line and during several forms of AKI in vivo [61]. Preliminary data suggest that the detrimental molecules ATP and ADP, released during cellular stress, are also targets of recAP as they are both rapidly converted into the cytoprotective adenosine.

Following these encouraging results, clinical pharmacology, safety and tolerability were evaluated in healthy volunteers. In this randomized, double-blind, placebo-controlled phase I clinical trial, single and multiple ascending intravenous doses recAP were well tolerated and could be administered without any safety concerns [62]. Subsequently, the efficacy of recAP is currently being investigated in an adaptive, multicenter, phase II clinical trial in patients with sepsis-associated AKI (NTC02182440). This two-stage trial will recruit a total of 290 patients. In the first part, the most effective dose out of three different doses of recAP will be determined, which will be further investigated in the second part of the study. While endogenous CCr during the first 7 days after start of administration of recAP is the primary endpoint, incidence and duration of RRT over 28 days and the subsequent occurrence of CKD will also be recorded, as well as various non-kidney-related clinical parameters.

## RRT in severe AKI: an overview

### Introduction

Untreated severe AKI in critically ill patients is associated with high mortality, and renal replacement therapies (RRTs) represent the cornerstone of the management of severe AKI. However, despite the dramatic evolution in technology for RRT, the mortality of AKI is still high. In 2015, a meta-analysis of 765 studies showed that the pooled incidence of AKI in hospital patients was 22% in adults and 14% in children and that the global mortality of AKI requiring RRT was 46% [30]. The aim of this brief narrative review is to describe the efficacy and clinical indications for different modalities of RRT in severe AKI patients.

### Modality: continuous RRT and intermittent hemodialysis

Different modalities of RRT have been and are used in the treatment of AKI, including continuous RRT (CRRT), intermittent hemodialysis (IHD), sustained low-efficiency dialysis (SLED) and peritoneal dialysis (PD). A worldwide survey [26] showed that CRRT was the most prevalent initial modality for AKI patients (80.0%), followed by IHD (16.9%), and PD and SLED (3.2%) (Table 3).

**Table 3:** Characteristics of CRRT, SLED and IHD

	CRRT	SLED	IHD
Modality	CVVH/CVVHDF/CVVHD	SLED/SLED-f	IHD/IHD-f
Duration per session	24 h	6–12 h	4 h
Frequency	24 h/day	3–6/week	3/week
Blood flow (ml/min)	100–200	100–200	250–350
Dialysate dose	20–25 ml/kg/h	100–300 ml/min	500–800 ml/min
Hemodynamic status	Stable	Possible stable	Unstable
Volume control	+++	++	+
Heparin dose	High	Moderate	Low

*CRRT* continuous renal replacement therapy, *SLED* sustained low-efficiency dialysis, *IHD* intermittent hemodialysis, *CVVH* continuous venovenous hemofiltration, *CVVHDF* continuous venovenous hemodiafiltration, *CVVHD* continuous venovenous hemodialysis, *SLED-f* sustained low-efficiency hemodiafiltration, *IHD-f* intermittent hemodiafiltration

Compared with other modalities, CRRT was considered as the predominant form of RRT in the ICU due to accurate volume control, steady acid–base and electrolyte correction, and the benefits on hemodynamic stability. However, although there might be some bias in patients selection, many randomized controlled trials (RCTs) and meta-analyses showed no difference in mortality between CRRT and IHD [63–65]. However, a meta-analysis [66] in 2013 reported that CRRT was associated with lower rate of dialysis dependence than IHD, and similar results were also found in a recent large cohort study [67]. Higher rate of dialysis dependence indicated that the real cost of IHD might be significantly higher than previously thought; in contrast, CRRT might be more cost-effective [68].

### Technique: hemofiltration, hemodialysis and hemodiafiltration

**Fig. 5:**
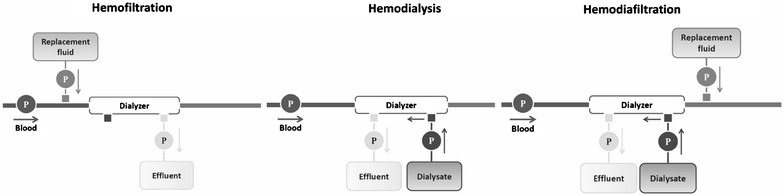
Schematic illustration of continuous hemofiltration, hemodialysis and hemodiafiltration circuits

If CRRT is being applied to the care of an ICU patient, the issue of preferred technique arises. As shown in Fig. 5, continuous hemofiltration (convective solute clearance), hemodialysis (diffusive solute clearance) and hemodiafiltration (combined convective and diffusive solute clearance) are the main solute clearance techniques in different kinds of CRRT. At this time, most clinicians appear to prefer hemofiltration or hemodiafiltration in critically ill patients with AKI, because of the belief that convective clearance might benefit patients by better removal of toxic inflammatory solutes, which are in the middle molecular range. Despite such beliefs, no studies have shown a convincing and sustained effect of continuous hemofiltration technique on circulating cytokine levels compared with continuous hemodialysis. Moreover, a recent meta-analysis [69] showed no effect of continuous hemofiltration on mortality and dialysis dependence in AKI patients compared with hemodialysis; in contrast, continuous hemofiltration appeared to shorten time to filter failure by 7 h. Thus, there is no level 1 evidence to guide clinicians in their choice of technique during CRRT, and there is some lower-level suggestive evidence that diffusion (hemodialysis) may be gentler on the filter and may therefore prolong circuit life.

### Less common techniques: slow low-efficiency dialysis (SLED)

As given in Table 3, SLED, a relatively new “hybrid” technology combining the properties from both IHD and CRRT, is a special form of intermittent dialysis with low dialysate and blood flow rates and prolonged duration. A recent meta-analysis [70], including 7 RCTs and 10 observational studies, reported that there was a mild trend toward improved survival in favor of SLED-treated patients with AKI (RR 0.86; 95% CI 0.74–1.00), although the evidence was weak because of a lack of significant differences when RCTs were considered separately. Nonetheless, there might be some potential advantages of SLED in general. First, SLED might lead to more rapid mobilization of patients and perhaps lead to shorter ICU stays and more rapid convalescence. Second, short and flexible duration of therapy might to some extent decrease the complications of RRT such as bleeding, hypotension, fluid overload as seen in other therapies like IHD. Third, a shorter duration of RRT might be associated with a lower rate of biofilm formation and circuit contamination.

### Peritoneal dialysis

In the past, PD has not been considered as the first choice of RRT for AKI in adults because of the low efficiency of solute clearance. However, there are now several RCTs focusing on continuous PD for AKI patients compared with IHD, CRRT or SLED, reporting similar mortality and kidney recovery [71–73]. A recent pooled meta-analysis also reported no difference in mortality between PD and extracorporeal RRTs [74]. Due to the lack of widespread use in developed countries and limited evidence, there is a need for better quality evidence in this important area.

### Conclusions

CRRT remains the most popular modality of RRT in ICU patients with severe AKI, but there is no evidence to support any benefits on mortality compared with other RRT modalities. However, compared with IHD, some large observational studies have reported higher rates of kidney recovery in CRRT-treated AKI patients, suggesting that IHD may adversely impact the process of renal recovery. The effect of SLED and PD on AKI needs to be better assessed and confirmed by high-quality studies. In clinical practice, individual-adjusted therapy should be recommended rather than focusing on any particular form of RRT. In this regard, there is a consensus that CRRT might be the optimal treatment for AKI patients with unstable hemodynamics or severe fluid overload. In contrast, IHD might be a reasonable choice when patients become stable or have left the ICU. The use of SLED may represent a reasonable alternative to CRRT in the ICU and a reasonable alternative to IHD outside the ICU.

### Positive fluid balance as an indication for RRT

Fluid administration is a key component of resuscitation strategies in the management of patients with hypotension and shock and can be envisioned in separate phases, permitting a clearer delineation of the therapeutic need [75]. In most instances, immediate resuscitation requires administration of adequate volumes of fluid and continued assessment and monitoring to determine improved hemodynamics and tissue perfusion [76]. Based on the surviving sepsis guidelines, the goals of therapy have included initial fluid boluses of 30 ml/kg followed by maintenance fluids to maintain adequate cardiac output and tissue perfusion. While several strategies have been tested for the fluid administration, it is unclear whether any approach is superior [77]. There are no criteria for deescalating fluid therapy and in practice fluids are often continued particularly when there is hemodynamic instability. A net result is accumulation of fluid over the course of therapy with resulting fluid overload. Several factors contribute to the fluid retention including an increased vascular permeability, alterations in the glycocalyx matrix and leakage of plasma proteins into the interstitial space with decreased oncotic pressure [78]. The process is further complicated if the kidney is affected by the underlying disease process or secondarily injured from the hypotension or nephrotoxic agents. The level of renal impairment may not be as evident as the SCr values are diluted by the fluid accumulation [11]. The resulting fluid overload, with thresholds of 10% excess from the admission weight, is associated with an incremental risk of mortality in patients with and without AKI [79]. Fluid overloaded patients have an increased risk of AKI [80, 81], and there is reduced renal recovery from AKI [82]. Several studies have shown that both the magnitude and persistence of fluid overload are associated with worse outcomes, suggesting that prevention and correction of fluid accumulation are modifiable risk factors to improve survival. Removal of fluid and optimization of fluid balance lower the risk and improve outcomes [80].

As the underlying disease process improves eventually the accumulated fluid is removed mainly through diuresis [83]. However, if the course is complicated by additional hits, fluid retention continues to accrue with deleterious consequences [84]. Salinas et al. [85] have shown that a computerized decision support for fluid management in burn patients led to reduce amount of fluid being used, lower fluid accumulation and improved survival. Similarly achieving negative fluid balance in patients with acute lung injury was associated with better outcomes [86]. It is thus imperative that fluid management strategies include a de-escalation phase to optimize fluid balance [87]. We have utilized CRRT to achieve and maintain fluid balance in these circumstances keeping hemodynamic balance in place [88–90]. We target an hourly fluid balance to maintain tissue perfusion and adjust volume administration and removal coupled with vasopressor and inotropic support for a comprehensive organ support. The CRRT system reduces the burden on the kidney, avoids the deleterious effects of aggressive diuretic use and permits fluid optimization by creating space for all the nutrition and drug delivery that is required. This fluid regulatory role of CRRT improves the time to weaning from ventilation and avoids the complications of prolonged fluid retention. Adjunctive CRRT therapy is a viable option to manage critically ill patients and should be considered for patients who have fluid accumulation particularly when renal function is impaired.

### IHD for shocked patients

After more than 20 years of an intense debate, the controversy still persists regarding the place of continuous or intermittent RRT (i.e., CRRT or IHD) to treat acute renal failure in ICU. The purpose of this controversy concerns mainly the tolerance of intermittent therapy for shocked patients and the associate complications like delayed renal recovery or death. For all that, KDIGO agree that the two methods may be used in ICU to treat AKI “as complementary therapies” [91]. The matter of debate remains for patients with hemodynamic instability requiring RRT. In this setting, international guidelines recommend the use of continuous therapy (KDIGO), while French guidelines consider the two methods equal [92].

Numerous old studies compared both methods. Most of them were non-randomized, retrospective trials and reported conflicting results. Many methodological biases preclude conclusive information. Nowadays, we know that hemodynamic tolerance can be significantly improved by using specific settings in IHD for critically ill patients [93]. These settings include a tailored net ultrafiltration amount with respect to the fluid balance, some dialysate modification (i.e., enriched sodium concentration and mild hypothermia) and duration above 4 h. Schortgen et al. [93] reported an improved hemodynamic tolerance using these settings. These results were confirmed in prospective randomized studies including patients with shock [64]. Keeping in mind that the number of patients randomized with shock is quite modest, no prospective study reports any hemodynamic adverse event using intermittent modality. To date, six prospective randomized studies have been published, some of them including shocked patients. Except one with group imbalance, all other did not find any significant difference of mortality of renal recovery comparing intermittent or continuous modality. These results are supported by several meta-analysis pooling the above-mentioned studies. Of note, continuous methods are not devoid of hemodynamic adverse events. In the RENAL study [94] comparing two doses of dialysis in ICU patients, the low-dose group experienced not less than 24% of arrhythmia, leading to hemodynamic instability.

Regarding renal recovery, the analysis is quite more difficult. There is no consensual definition and the evaluation relies usually on ICU or hospital discharge, which may be relatively short. Usually, the definition is based on RRT dependency. However, delayed renal recovery and death represent two competitive risks. While prospective comparative studies are negative with respect to renal recovery, retrospective studies report higher renal dependency with intermittent modality. The non-randomized design leads to imbalance between groups. Severe patients with high mortality are treated with CRRT, while IHD is dedicated to less severe patients. Thus, the risk to become dialysis dependent is increased in the group with the lowest mortality (i.e., IHD group).

Finally, what may best explain the discrepancy between the two modalities is the practice across the world. Based on a questionnaire, it appears that IHD is usually prescribed by nephrologists and monitored by dialysis nurses, while CRRT is under the authority and the monitoring of intensive care team [95]. With this organization of care, we can guess that the availability of IHD as well as the experience of ICU team with this modality is unsuitable for most ICU patients whatever the quality of IHD.

To conclude, we could combine the two recent guidelines [91, 94] in the same sentence: “Continuous and intermittent RRT techniques can be used equally, as complementary therapies, taking into account their availability and the experience of the team.”

## HCO membranes in sepsis

### Introduction

The release of pro- and anti-inflammatory cytokines from activated immune cells is a key feature of inflammation and is vital for pathogen clearance and attenuation/recovery of tissue damage. Uncontrolled or imbalanced release of such cytokines can, however, be harmful. In fact, an overwhelming inflammatory response to surgery, trauma and infections is a major cause of organ damage and mortality in critically ill patients [96]. Cytokine removal by extracorporeal blood purification has been suggested a potential therapeutic option to improve outcomes in septic patients [97]. Such blood purification techniques include high-volume hemofiltration, plasma adsorption, plasma filtration, combined plasma filtration and adsorption and high cutoff (HCO) hemofiltration and hemodialysis. Of these techniques, HCO hemofiltration/hemodialysis appears particularly efficient to achieve high cytokine clearance [98]. We describe the biochemical effects of this technique and its potential clinical effects.

### HCO membrane characteristics

Membrane cutoff is defined by the molecular weight (in kDa) of molecules with a sieving coefficient (SC) of 10% across the membrane. Conventional membranes have a cutoff of approximately 30 kDa, i.e., only about 10% of middle molecular weight cytokines (e.g., TNF-alfa, 26 kDa) would theoretically pass such filters. In reality, however, the SC is significantly lower, which means that cytokine removal during standard RRT is negligible [98]. In contrast, HCO membranes have a clinical cutoff of 60–100 kDa and a pore size approximately twice as large as that in conventional membranes. A systematic review of ex vivo studies concluded that HCO hemofiltration achieved greater median clearance of IL-1β (1.4-fold), IL-6 (tenfold) and TNF-α (60-fold) than standard hemofiltration. Furthermore, with the exception of TNF-α, HCO hemofiltration greatly enhanced cytokine clearance in animal and human experiments [99, 100].

Cytokine clearance is dictated not only by molecular weight and membrane characteristics but also, as confirmed by previous ex vivo studies, by the intensity and mechanism (diffusion versus convection) of solute clearance [101, 102]. Compared to hemodialysis (diffusive clearance), higher clearance of IL-1ra, IL-1β, IL-6, IL-8 and TNF-α is achieved with hemofiltration (convective clearance). Increased cytokine clearance with higher ultrafiltrate/dialysate flow rate can also be expected. In addition to improved cytokine removal during hemofiltration, the concomitant loss of essential proteins, such as albumin, has been a concern. In fact, albumin clearance can amount to 10 ml/min during hemofiltration [102]. Such albumin losses can, however, easily be replaced by infusion of albumin solutions.

### Cytokine removal via HCO membranes: clinical effects

So far, only a few small studies have been explored the clinical utility of HCO membranes in septic patients (Table 4). Cytokine clearance and illness severity were quantified in 24 patients with septic AKI randomized to continuous venovenous hemofiltration (CVVH) or continuous venovenous hemodialysis (CVVHD) [103]. Additionally, the clinical and biochemical effects of ultrafiltration rate and dialysate flow rate were explored. Compared to CVVHD, greater IL-6 clearance was achieved with CVVH. Irrespective of modality, higher flow rates led to greater IL-6 and IL-1ra clearance. Overall, APACHE II and multiorgan dysfunction syndrome score decreased after 24 h, however, without a detectable difference between the CVVH and CVVHD groups.

**Table 4:** Cytokine clearance, albumin clearance and clinical effects of renal replacement therapy using high cutoff membranes

First author, year	*N*	RRT modality	Qf or Qd (l/h)	Cutoff^a^ (kDa)	Cytokine clearance	Albumin clearance	Clinical effects
Morgera et al. [103]	24	CVVH versus CVVHD	Qf 1 versus 2.5 Qd 1 versus 2.5	60	Greater IL-1ra clearance with CVVH. Increased Qf or Qd increased IL-6 and IL-1ra clearance	Highest with CVVH 2.5 l/h	Overall decrease in APACHE II and MODS scores. No difference between groups
Morgera [235]	30	CVVH	Qf 2.5	30 versus 60	Greater IL-6 and IL-1ra clearance with 60 kDa-filter	Plasma albumin levels not affected by filter cutoff	Reduced noradrenaline requirements with 60 kDa-filter
Haase et al. [104]	10	IHD	Qd 18	20 versus 60	Greater IL-6, IL-8 and IL-10 clearance with 60 kDa-filter	Plasma albumin levels not affected by filter cutoff	Trend toward increased mean arterial pressure and reduced vasopressor requirements with 60 kDa-filter

*RRT* renal replacement therapy, *Qf* ultrafiltration rate, *Qd* dialysate flow rate, *CVVH* continuous venovenous hemofiltration, *CVVHD* continuous venovenous hemodialysis, *IHD* intermittent hemodialysis, *APACHE* acute physiology and chronic health evaluation, *MODS* multiorgan dysfunction syndrome

^a^Estimated in vivo membrane cutoff

In a randomized controlled trial, 30 patients with septic AKI were allocated to HCO CVVH (*n* = 20; membrane cutoff 60 kDa) or conventional CVVH (*n* = 10; membrane cutoff 30 kDa) using post-filter replacement volumes of 2.5 L/h in both groups [9]. At 48 h, decreased plasma levels of IL-6, IL-1ra and CRP was observed in the HCO group but not in the conventional group. Furthermore, patients treated with HCO CVVH had significantly lower SAPS II score and vasopressor requirements after 48 h suggesting a clinical benefit of cytokine removal.

A phase 1 crossover trial compared a HCO filter with a conventional filter during IHD in 10 septic AKI patients [104]. A greater decrease in plasma IL-6, IL-8 and IL-10 was observed after 4 h of HCO-IHD, whereas no difference in IL-18, urea and albumin removal was found. Interestingly, there was a trend toward increased MAP and reduced vasopressor requirements after a 4-hour treatment with HCO-IHD.

A likely link between sepsis-induced release of inflammatory mediators (e.g., cytokines), activation of apoptotic pathways and organ injury has been proposed [105, 106]. Whether cytokine removal mitigates this response and translates into clinical benefits should therefore be explored. Recently, a randomized controlled trial (ClinicalTrials.gov Identifier: NCT00912184) completed recruitment of 76 patients with the aim to compare vasopressor requirements during HCO (100 kDa) CVVH or standard (30 kDa) CVVH. In a subset of patients enrolled in that trial, pro-apoptotic plasma activity was compared between the two groups [107]. At baseline, apoptotic activity in these AKI patients’ plasma was evident by DNA fragmentation, caspase-3 activity and phosphatidylserine exposure on cell membranes. After 24 h, significantly less phosphatidylserine exposure was demonstrated in the HCO group, whereas no difference in DNA fragmentation or caspase-3 activity was found. Over a 3-day assessment period, no robust changes in apoptotic activity were seen in either group. Based on these findings, the effect of cytokine removal on apoptosis and organ injury remains uncertain and needs to be further explored.

### Conclusion

Blood purification via HCO filters has been safely used in septic AKI patients to effectively remove cytokines from the circulation. Hemofiltration increases cytokine clearance more than hemodialysis but also leads to greater albumin losses. Clinical benefits of blood purification via HCO filters in septic patients have been suggested but need to be determined in larger trials.

### Vascular access sites for acute renal replacement in ICUs

The treatment of severe AKI with RRT often requires to obtain a central venous access. Non-tunneled, non-cuffed, temporary, relatively large central venous catheters (CVC) are used for this purpose in the critical care environment. A good vascular access site should reduce the risk of immediate mechanical complications during insertion, limit the risk of late infectious or thrombotic complications once the catheter is inserted and provide an adequate flow to perform RRT during the course of AKI.

The subclavian veins should not be used when possible, as there is an increased risk of thrombosis when large catheters are inserted in a small vein. The right jugular vein has been historically considered the gold standard for vascular access in this context and the femoral vein should be avoided, or considered in the last resort for emergent situations. This poor reputation was based on a seminal study in which the risk of catheter-related bloodstream infection increased exponentially after one week among patients requiring acute hemodialysis [108]. Of note, this study was not randomized and performed outside the intensive care setting. Patients in the medical wards, however, differ in many respects from critically ill patients, raising the question of whether these recommendations can be extrapolated to critically ill patients.

To investigate which vascular access was best for acute RRT in the ICU, our group conducted the first large randomized multicenter study comparing internal jugular and femoral vein catheterization in the ICU. The CATHEDIA study [109–112] was aimed to compare the jugular and femoral sites for:

1. the risk of catheter infection
2. the risk of mechanical complications
3. the risk of thrombosis
4. the risk of catheter dysfunction and the quality of RRT.

In addition, the epidemiology of the infectious risk according to the type of RRT (e.g., IHD versus continuous venovenous hemodiafiltration) and the case of the second catheter were investigated. The key findings of the CATHEDIA trial are summarized in Table 5.

**Table 5:** Main results of the CATHEDIA trial

Refs.	Design	Outcome	Highlights
[2]	RCT, parallel	Catheter infection	The risk of catheter infection inserted in FEM and JUG is similar
	RCT, parallel	Catheter infection	JUG site may be preferred in obese patients
	RCT, parallel	Thrombosis	The risk of thrombosis is similar in FEM and JUG is similar
	RCT, parallel	Severe mechanical injury	Without ultrasound guidance, FEM is safer than JUG
[3]	RCT, parallel	Catheter dysfunction	The risk to dysfunction is similar in FEM and JUG is similar
	Cohort	Catheter dysfunction	Right side of the body should be preferred for JUG
	RCT, cluster	Dialysis quality	Urea Reduction Ratio is similar in FEM and JUG
	Cohort	Dialysis quality	For blood flow >200 ml/min, jugular is better
	Cohort	Dialysis quality	Length for FEM catheter should be >25 cm
[4]	Cohort	Catheter colonization	The risk of infection does not increase overtime with hemodialysis
	Cohort	Catheter colonization	The risk of infection increases overtime with hemodiafiltration
[5]	RCT, crossover	Catheter infection	The risk of catheter infection inserted in FEM and JUG is similar
	RCT, crossover	Dialysis quality	Urea Reduction Ratio is similar in FEM and JUG
	RCT, crossover	Catheter dysfunction	The risk to dysfunction is similar in FEM and JUG is similar

Overall, the results of the CATHEDIA study suggest that the best vascular access choice may depend on several factors independent from the patient such as the physician experience and the availability of ultrasound guidance. In the large majority of cases, the femoral and jugular accesses will carry a similar risk of complications and similar dialysis quality. There are some exceptions however, in which the intensivist may want to prefer one site over the other: The right internal jugular insertion site may be preferred to deliver the best RRT dose if the prescribed blood blow is higher than 200 ml/min, the femoral site should be avoided if the BMI > 28, if the femoral site is contaminated or if the patient is ambulatory; the jugular site should be avoided in case of tracheostomy or if the site is contaminated.

The management of dialysis catheter represents an important factor for the success of ICU RRT. The type of catheter and catheterization procedures, especially in the insertion site and catheter maintenance (flushes, locks), affects the quality of RRT and the risk of catheter dysfunction. The epidemiology of the catheters used for RRT is very similar to the more studied epidemiology of the catheters used for administrating drugs, although the rate of thrombosis seems lower in RRT CVC, possibly due to anticoagulation. Therefore, the same bundle of care needs to be implemented to limit the risk of potentially severe complications. This includes infection control procedures and checklists, learning and teaching safer vascular access by the use of ultrasound-guided insertion (real time), removing unnecessary catheters and optimal skin disinfection and CVC care with alcoholic 2% chlorhexidine or alcoholic povidone–iodine in case of contraindication to chlorhexidine use. Of note, catheters should not be removed after a predetermined amount of time to prevent the risk of infection [109].

During the last decade, new evidence-based data regarding vascular access have emerged. We hope these findings will inform intensivists and contribute to avoid potentially preventable healthcare-associated complications while providing better quality of care to this severe subset of the ICU population.

### Acute renal failure as a witness of systemic diseases

In 10–20% of patients with ARF, not related to obstruction or hypovolemia, a systemic disease is the cause of AKI, affecting mainly small vessels and glomeruli. Usually, an associated acute tubular necrosis (ATN) is present. Macro-proteinuria, albuminuria, hematuria and extrarenal signs, affecting skin, joints, neurons or lung, should alert the clinician that the clinical presentation is quite different from the usual AKI seen in ICU, that is ATN. The main diagnoses are rapidly progressive GN (RPGN), thrombotic microangiopathies (TMA), cholesterol crystal embolism and catastrophic antiphospholipid syndrome [36]. For RPGN, an extensive immunologic screening, looking for ANCA, anti-GBM antibodies, ANA and anti-DNA antibodies, cryoglobulinemia and complements C3 and C4, is needed. A renal biopsy may be required [36] to determine the appropriate treatment, which should be started rapidly. For TMA, anemia, reticulocytes, schistocytes, increase in LDH, low haptoglobin levels and low platelet count are found. The diagnostic screening includes stool culture to detect Shiga-toxin-producing E coli, PCR analysis for Shiga-toxin detection, plasma ADAMTS-13 determination, as well as alternate pathway of complement exploration (C3, C4, facteur H, I and MCP, factor B) and antiphospholipid antibodies.

RPGN are characterized by an acute glomerular extracapillary proliferation and fibrin deposition, combined with a rapidly progressive renal failure. By immunofluorescence, different patterns are observed: linear deposition of IgG along the glomerular basement membrane in Goodpasture syndrome, where pulmonary hemorrhage is life-threatening; granular deposition of IgG and C3 in lupus disease, cryoglobulinemia, and endocarditis; IgA deposition in Henoch–Schonlein disease; and no significant immune deposition in ANCA-associated vasculitis. An aggressive treatment is needed, with high doses of steroids, plasma exchanges and cyclophosphamide, to prevent life-threatening complications and to improve renal prognosis. The poorest renal and patient prognosis is associated with a SCr at entry greater than 500 µmol/l, a need for hemodialysis and crescentic lesions in 100% of the glomeruli. In ANCA-associated vasculitis, plasma exchanges were shown to improve renal function and renal prognosis but not patient survival when compared to high doses of steroids [113]. More recently, rituximab was reported to give similar results when compared to cyclophosphamide in mild-to-moderate forms of the disease.

In TMA with acute renal failure, different mechanisms and diseases are possible: Shiga-toxin-induced hemolytic uremic syndrome (HUS), atypical HUS, more rarely thrombotic thrombocytopenic purpura (TTP) with low ADAMTS-13 activity. It may also be related to drug-related TMA (bevacizumab, calcineurin inhibitors, gemcitabine, mitomycin C), systemic infection, systemic cancer, severe preeclampsia and HELLP syndrome, malignant hypertension, autoimmune disease (systemic sclerosis, lupus, CAPS) or hematopoietic stem cell or organ transplantation. The differential diagnosis is sometimes difficult in adults. Clinical signs are numerous, including renal failure, hypertension, abdominal pain and diarrhea, headaches and seizures, myocardial infarction and cardiomyopathy. When performed, the renal biopsy shows microthrombotic lesions in glomerular capillary and/or small arterioles, double contour pattern, and in the most severe forms, areas of cortical necrosis. In most of the cases, plasma therapy has to be started rapidly, usually with 60 ml/kg/day plasma exchanges until normalization of platelets count. If TTP is diagnosed, rituximab should be considered in the absence of rapid normalization of platelet count. If complement-mediated HUS is diagnosed, eculizumab should be administered. In cases of Shiga-toxin-induced HUS, there is no evidence that plasma exchanges, steroids or eculizumab are useful [114], although some case reports suggest that eculizumab should be given in the most severe forms [115]. In any case, it is important to strictly control hypertension, since hypertension per se has a deleterious effect on the microvascular lesions and plays an aggravating role in HUS.

In conclusion, ARF in ICU is not always related to acute tubular lesions. It can be also related to systemic diseases that have to be recognized rapidly since specific treatments are available that change both the renal and patient prognoses.

## Liver and kidney: a relationship

### Definitions

Traditionally all patients with renal dysfunction and liver disease were classified as having the dreaded diagnosis of hepatorenal failure and labeled with a dismal prognosis and not offered further therapeutic interventions—thankfully things have and continue to change and we have become more elegant in both our descriptions and management of patients with renal dysfunction.

The new definitions as described by Wong and Angeli, respectively [116, 117], clearly recognize the importance of the utilizing RIFLE and ADQI definitions in delineating hepatorenal failure (HRF) types 1 and 2. Acute kidney injury (AKI) in cirrhosis is described as a rise in creatinine of >50% from baseline or a rise of >26.5 µmol/l in 48 h, with type 1 HRF being a specific form of AKI. Chronic kidney disease (CKD) is defined as a glomerular filtration rate (GFR) of <60 ml/min for greater than 3 months and specifies that HRF type 2 is a specific form of CKD. A further category of acute of chronic kidney disease (AoCRF) is a rise in creatinine of >50% from baseline or by greater than 26.5 µmol/l in less than 48 h in a patient with a GFR < 60 ml/min for greater than 3 months.

### Pathophysiology

The classic description for the development of HRF is based on the development of arterial vasodilation and splanchnic vasodilation and effective central blood volume depletion. This results in activation of vasoconstrictor systems (angiotensin, aldosterone), altered renal autoregulation, intra-renal vasoconstriction and sodium retention [118]. Contributory factors in the CKD of cirrhotics are parenchymal renal disease, diabetic, hypertensive and immune-mediated pathologies (Ref). Large-volume ascites and associated intra-abdominal hypertension may decrease renal perfusion pressure result in a further insult (AoCRF). In a subgroup of cirrhotics, pulmonary venous hypertension results in central volume overload and elevated right-sided pressures may also contribute to both CRF and AoCRF (refs). Pulmonary arterial hypertension becomes significant when pressure volume overload develops.

### Diagnostic difficulties

One of the concerns of a cutoff creatinine level to define AKI in cirrhosis is the failure of creatinine to equate to GFR. Correlation between eGFR and gold-standard GFR (EDTA or iohexol) is poor due to a combination of decreased creatine production and decreased muscle mass. Recent studies demonstrated that only 30% of patients with a gold-standard GFR of <60 ml/min were identified using calculated formulae. Normal creatinine in a cirrhotic was likely being in the region of 65 µmol/l as compared to the standard normal range [119].

The diagnostic criteria for HRF within the AKI model are that of diagnosis of AKI, no response to intervention over 48 h, absence of shock, no recent use of nephrotoxins, contrast media and no macroscopic signs of parenchymal disease (no proteinuria of >500 mg/day, no microhematuria and normal renal ultrasound). Using these standards HRF is likely to a rare diagnosis

Various biomarkers were examined in a recent study by Belcher et al. [120], NGAL, IL-18, KIM-1, L-FABP, proteinuria and urinary sodium all had diagnostic utility to separate pre-renal, HRF and acute tubular necrosis. Area under the curve was optimal at >0.7 for NGAL and IL-18 and proteinuria but only 0.56 for urinary sodium. The presence of low-level proteinuria (PCR > 30) has also been shown to be highly predictive of development of AKI [119].

The underling etiology of AKI has been shown by two studies to clearly impact on prognosis; the worst 3-month survival being seen for HRF (15%), while hypovolemia was 46% and infection related 31% [121] and similar data seen in [122]

### Management

Management of HRF has been described elegantly in the paper of Angeli et al.; stage 1 AKI should result in the removal of putative nephrotoxins, treatment of hypovolemia and sepsis, hyponatremia should always result in diuretic withdrawal. If AKI progresses to stages 2 and 3, diuretics are discontinued if this has not already occurred, and albumin should be administered at 1 g/kg for 2 days. If the patient then meets criteria for HRF, vasoconstrictor therapy should be administered. Volume should be administered if the patient is though deplete—a simple statement and a complex clinical decision. Clinicians are poor at delineating volume status, and the use of echocardiography should be considered to define volume status and avoid volume overload or excessive fluid administration, both of which are as detrimental as volume depletion.

Instituting vasoconstrictor therapy only at stages 2 and 3 may be questioned given the work of Krag et al. [123] showing beneficial effects on eGFR, renal blood flow, urinary sodium clearance and water clearance when administered to cirrhotics with ascites but no AKI. These data along with that of greater chance of therapeutic response being seen with lower creatinine levels would argue for earlier consideration of vasoconstrictors, albeit it recognizing the potential side effects of potent vasoconstrictor. Albumin is proposed for all patients with AKI and has benefit in SBP sepsis, although this effect has not been seen in other septic etiologies [124].

The data to support the use of vasoconstrictor and albumin therapy is supported with regard to reversal of HRF by a Cochrane review, and data from the Terlipressin Study Group showed reversal of HRF is seen to be affected by treatment group, while survival is related to etiology of alcoholic hepatitis, MELD score and serum creatinine [125]. The choice of vasoconstrictor therapy is normally that of terlipressin, with a starting dose of 0.5 mg 6 hourly and rising to 1 mg 6 hourly. Duvoux et al. showed a response to norepinephrine, and a RCT demonstrated equivalence when comparing norepinephrine and terlipressin, with the predictors of outcome being creatinine clearance at enrollment, mean arterial pressure and renin level [126]. Glucocorticoid therapy with or without *N*-acetylcysteine (NAC) for the treatment of alcoholic hepatitis was associated with decreased HRF in the NAC limb [127].

Other therapeutic options should consider the detrimental effect of elevated intra-abdominal pressure (IAP) in decreasing renal perfusion pressure and contributing to AKI. Treatments may be to increase mean arterial pressure or low-volume paracentesis to decrease intra-abdominal pressure. Data from Umgelter et al. [128] showed a decrease in creatinine following low-volume paracentesis. When draining ascites caution should be exercised to prevent further central hypovolemia with associated with a decrease in IAP and subsequent splanchnic vasodilation, this can be achieved by either albumin therapy or terlipressin [129].

In patients who do not respond to vasoconstrictor therapy, consideration should be given to undertaking renal replacement therapy (RRT). Early institution of RRT has been suggested to improve outcome and in cirrhosis this is especially pertinent given the failure of creatinine to reflect GFR. Furthermore, early RRT allows control of serum sodium and avoidance of critical hyponatremia which may, along with hyperammonemia, contribute to significant deterioration in conscious level (refs).

### Outcome

Development of AKI or AoCKD is clearly associated with increased mortality, and the CLIF organ failure score includes creatinine along with bilirubin, coagulation, circulatory and respiratory parameters [130]. The subsequently refined CLIF-C score incorporates WBC and age [131]. Predictors of mortality (30 day) in cirrhotics with AKI have been shown to be mean arterial pressure, severity of liver disease as measured by MELD score, spontaneous bacterial peritonitis (SBP) and number of organs failing [132].

### ARF as a witness of cardiac arrest

**Fig. 6:**
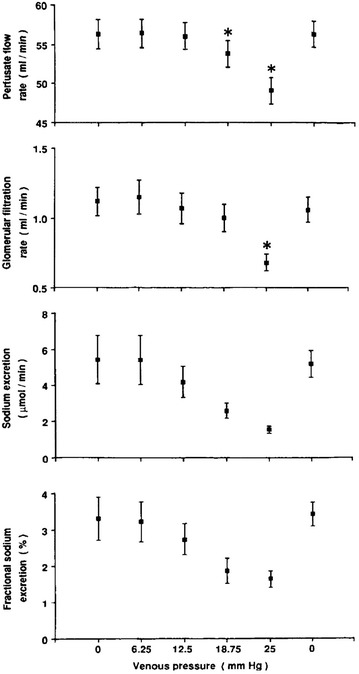
Decrease in perfusate flow, glomerular filtration and sodium excretion after stepwise increase in venous pressure in kidneys perfused at constant arterial pressure (from 2)

Acute heart failure patients have a high risk of developing renal failure under the type 2 cardiorenal syndrome (i.e., acute heart failure affecting renal function). Furthermore, acute cardiorenal syndrome has been associated with poor outcome in acute heart failure patients. Systemic hemodynamic has long been thought to play a central role in the worsening renal function associated with acute decompensated heart failure (ADHF) [133]. Historical physiological studies have emphasized the role of venous congestion. Increasing central venous pressure while maintaining arterial pressure is associated with a drop of renal blood flow, drop of GFR and anti-natriuresis [134] (Fig. 6). More recently, CVP was shown to be associated with a risk of WRF in ADHF patients, while cardiac index or arterial pressure was not. Legrand et al. also observed that CVP was associated with a risk of worsening renal function in patients with severe sepsis, especially when CVP rose above 12 mmHg [135]. Herrler et al. further highlighted the role of increase compartmental pressure in the kidney in showing that capsular removal induced pressure relief and prevented functional and structural renal impairment after renal ischemia–reperfusion [54]. The impact of venous congestion the kidney can, however, lie way beyond the alteration of renal function (detected as a decrease in eGFR or a rise in serum creatinine). In this line, because it is the easiest and most available biomarker of renal injury, drop of GFR may only represent the tip of the iceberg of renal consequences of venous congestion. The use of recently developed biomarkers of renal injury (especially tubular injury) has allowed to identify some degree of renal injury in heart failure. However, renal injury biomarkers have failed in many studies to identify patients who later had a drop of GFR due to the complex relationship between renal injury and glomerular function in these patients [133]. Transient venous congestion has also been shown to alter the microcirculation and induce endothelial injury and local inflammatory response [136]. Hypervolemia was shown to degrade the glycocalyx at the surface of the endothelial layer, an essential compound of the microvascular function influencing permeability. Release of natriuretic peptide might play a role in this degradation since infusion of ANP in animals degraded the glycocalyx layer independently of changes of intravascular volume. A key component of the venous congestion in acute heart failure patients appears to be the excess of intravascular volume, which has been found to range from +9.5 to +107% of normal value in ADHF on admission [137]. Reasons for intravascular volume result from progressive retention of water and sodium due to anti-natriuresis in such patients. Several factors contribute to intense sodium reabsorption ADHF patients. Among them, decrease in renal blood flow contributes to increase in sodium reabsorption at the proximal tubules level. Maintenance of GFR lies on increase of filtration fraction with hemoconcentration in the efferent arteriole and peritubular capillaries. Increase in protein concentration in peritubular capillaries will then promote passive sodium and water reabsorption in proximal tubules. Activation of the renine–angiotensine–aldosterone system is another factor promoting sodium tubular reabsorption. Interestingly, use of loop diuretics can promote RAAS activation through decrease in chloride concentration in cells of the macula densa leading to renin secretion.

Therapeutic strategies to control sodium and fluid balance in ADHF aim at limiting venous congestion and renal injury. In this line, some degree of hemoconcentration with rise in hematocrit and slight decrease in GFR has been associated with better outcome in ADHF [138]. Furthermore, failure to increase diuresis and to control fluid balance using loop diuretics has been associated with poorer outcome in these patients. Interestingly, GFR does not appear to be a predictive factor to loop diuretics resistance, suggesting that altered intra-renal hemodynamics and tubular dysfunction might be involved. Association of diuretics can overcome this resistance to diuretics. Thiazide diuretics inhibit distal tubules sodium reabsorption. Acetazolamide through proximal tubules sodium reabsoption inhibition and mineralocorticoids receptors inhibitors are also to be considered although data in ADHF patients are lacking. Finally, interest of ultrafiltration has long been emphasized in these patients, but review of its use is out of the scope of this manuscript. Future studies should better determine whether fluid control strategies may protect the kidney form injury and failure in ADHF and may improve global outcome in these patients in modulating injury pathways through organ cross talks.

### Acute renal failure as a witness of abdominal hypertension

**Fig. 7:**
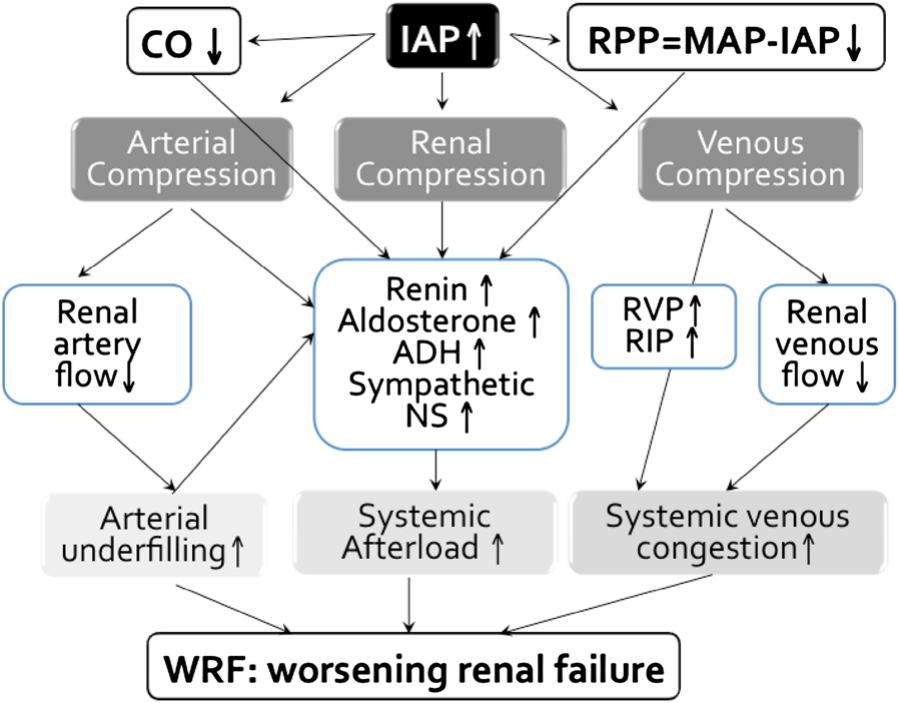
Possible mechanisms leading to worsening renal failure during increased intra-abdominal pressure. *ADH* antidiuretic hormone, *CO* cardiac output, *IAP* intra-abdominal pressure, *MAP* mean arterial pressure, *NS* nervous system, *RIP* renal interstitial pressure, *RPP* renal perfusion pressure, *RVP* renal venous pressure

**Fig. 8:**
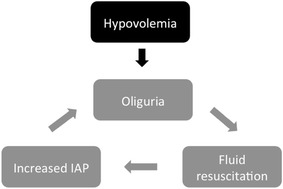
Vicious cycle leading to fluid accumulation and worsening renal failure in patients with hypovolemia or sepsis

A recent meta-analysis confirmed that intra-abdominal hypertension (IAH), defined as a sustained increase in intra-abdominal pressure (IAP) above 12 mmHg, and abdominal compartment syndrome (ACS), defined as a sustained increase in IAP above 20 mmHg with new onset organ failure, occur commonly in critically ill patients [139]. Both are independently associated with morbidity (organ dysfunction) and mortality [139, 140]. Around 30% of critically ill patients have IAH on admission, and this is mainly related to fluid overload, while around 5% develop full-blown ACS [139]. In order to establish a diagnosis, IAP needs to be measured, the gold standard being via the bladder [140]. Mortality of ACS is high when left untreated. The kidney is an encapsulated organ, located in the retroperitoneal space of the abdominal compartment that is especially vulnerable to the deleterious effects of increased IAP due to the anatomical position and blood supply. The kidney is often the first organ that fails when IAP is increased and can be considered the canary in the coalmine for IAH and ACS [141]. Already in 1873, Wendt E.C. from Germany stated “The higher the abdominal pressure the less the secretion of urine,” IAH has been associated with renal impairment for over 150 years, but it is only recently that a clinically recognized relationship has been found [141]. Several animal studies have provided some insights into the mechanism of renal dysfunction in IAH [142]. The adverse effects of elevated IAP on renal function can already occur at lower levels of IAP, before the development of overt ACS [142]. An increasing number of large clinical studies have identified that IAH is independently associated with renal impairment and increased mortality [142]. The mechanisms of renal impairment are not fully understood, but are probably multifactorial: reduced renal blood flow, reduced cardiac output and increased systemic vascular resistance together with alterations in hormonal (plasma renin activity) and neurogenic factors (Fig. 7). Fluid overload may trigger a vicious cycle leading to further kidney and venous congestion (especially in patients with sepsis and capillary leak with secondary IAH) as shown in (Fig. 8) and should be avoided [87]. Hence, diuresis is not a good parameter to guide fluid resuscitation in critically ill patients with IAH. Elevated IAP significantly decreases renal venous and arterial blood flow leading to renal dysfunction and failure [142]. Oliguria usually develops at an IAP of 12–15 mmHg and anuria at 25–30 mmHg in the presence of normovolemia and at lower levels of IAP in patients with hypovolemia or sepsis or under mechanical ventilation with high levels of positive end-expiratory pressure [141]. RPP and renal filtration gradient (FG) have been proposed as key factors in the development of IAP-induced renal failure [141].

$${\text{RPP}} = {\text{MAP}} - {\text{IAP}}$$

where MAP = mean arterial pressure

$${\text{FG}} = {\text{GFP}}{-}{\text{PTP}} = \left( {{\text{MAP}}{-}{\text{IAP}}} \right){-}{\text{IAP}} = {\text{MAP}}{-}2 \times {\text{IAP}}$$

where GFP = glomerular filtration pressure, PTP = proximal tubular pressureThus, changes in IAP have a greater impact upon renal function and urine production than will have changes in MAP. It should not be surprising, therefore, that decreased renal function, as evidenced by the development of oliguria, is one of the first visible signs of IAH. Conversely, therefore it behooves us as clinicians to be cognizant of the elevated IAP and its effect on renal function is often the first sign of impending ACS. Other important issues to remember will be further discussed. The pre-renal azotemia seen in IAH is unresponsive to volume expansion to a normal CO, dopaminergic or inotropic agents or loop diuretics. The impairment in renal function produced by increased IAP seems to be a local phenomenon caused by direct renal compression and is not solely related to cardiac output. Renal function may be improved by paracentesis of the ascitic fluid and subsequent reduction in the IAP. Prompt reduction of IAP has dramatic beneficial effect on UO in patients with primary and secondary ACS after trauma. Within the capsule of the kidney itself, local hematoma formation may have an adverse affect on tissue perfusion causing a local renal compartment syndrome. The interactions between different compartments have been referred to as the polycompartment syndrome, and [143] within this concept the compliance may play a major role [144]. Intriguingly, in advanced heart failure—presumably because of low renal perfusion—the kidneys are extremely sensitive to even small elevations in IAP (8–10 mmHg) [145]. Moreover, decreasing IAP in such cases, through ultrafiltration or paracentesis, can dramatically improve renal function. Within this regard, it is important to consider IAP as a missing link in patients with congestive heart failure developing worsening kidney function. This condition has been termed as CARS, cardio-abdominal renal syndrome [145]. The best prevention of AKI is prevention of IAH, and the best treatment is treatment of IAH/ACS. The World Society of the Abdominal Compartment has suggested several treatment options, and they can be summarized in four major therapeutic approaches: First, improvement of abdominal wall compliance, second, evacuation of intraluminal contents, third, evacuation of abdominal fluid collections and finally, correction of capillary leak and positive fluid balance [140].

### RRT management: optimal timing

Deciding when to initiate dialysis in a critically ill patient remains one of the most challenging questions in the management of critically ill patients. Although several approaches have been offered, there is considerable variation in when dialysis is offered in the ICU setting [146]. There is widespread recognition that timing of initiation of dialysis is a key area that needs additional research [147]. Meta-analysis of trials [147] looking at timing of dialysis suggests that there is a signal for improved outcomes with earlier starts; however, it is not clear what is the definition of early as there are no standard criteria for evaluating timing of dialysis [148–151]. Several factors have contributed to our lack of standardization in this field. Our current approach to offering dialysis is strongly conditioned by our experience with RRT in patients with end-stage renal disease (ESRD) where dialysis is not offered unless there is evidence that GFR is 5–10 ml/min and there is evidence of complications of uremia [152]. Consequently, our standard indications for RRT reflect severe derangements in renal function including marked acidosis, uremia, severe electrolyte problems or diuretic resistant fluid overload. A second factor is that in critically ill patients the heterogeneity of presentation with multiple organ failure the kidney is often overlooked and less demanding for attention when the focus is on improving and maintaining cardiac performance. Thirdly, the consequence of process of care including aggressive resuscitation may impose significant demands on the kidney wherein the normal excretory capacity may be overwhelmed. Additionally, underlying comorbidities including CKD and heart failure further limit the range of renal capacity. Finally, drug and nutritional administration contribute to the demand for fluid removal to maintain fluid balance. The dissimilarities of the critical care environment from the stable ESRD patient thereby highlight the need for different strategies for application of RRT. Protocols have been proposed to standardize decisions for RRT initiation but have not been formally evaluated [148, 153]. Whereas RRT is by necessity offered only as a final resort in ESRD, its application in ICU patients should be tailored to the need [154]. We have proposed a simple model to address this conundrum in considering the relationship of the demands being placed on the kidney with the underlying capacity [155]. Using this framework, one can characterize patients into four groups (Table 6) and develop systematic strategies to address each. When demand exceeds capacity, it becomes necessary to offer RRT to support renal function. The magnitude of the demand capacity mismatch can be quantified and utilized to guide therapy initiations and discontinuation. We are currently testing a model to validate these concepts. Several ongoing studies are also addressing this issue with different strategies. It is anticipated that the concerted effort in this area will ultimately provide new data to improve our management of these patients.

**Table 6:** Model to initiate and stop dialysis based on assessment of demand and capacity

Demand	Capacity	Example	Action
High	Normal	High catabolic state High nutritional loading Poisoning	Reduce demand if possible Monitor for support renal support
High	Low	Decreased GFR from AKI	Renal support Reduce demand if possible
Normal	Low	CKD Non-catabolic AKI	Add renal support if necessary to maintain steady state
Low	Low	Malnutrition and wasting CKD	Assess for nutritional state and add renal support if necessary

## Dialysis dose in AKI

### Introduction

AKI is a common complication of critical illness and is associated with high morbidity and mortality. Due to the lack of effective drugs, when AKI becomes severe enough, RRT is considered the treatment of choice. In the past 15 years, many studies have focused on the intensity (dose) of RRT in AKI patients. The aim of this brief narrative review is to describe the concept and impact of dialysis dose of RRT in AKI patients.

### Dialysis dose of RRT in AKI

In 2000, a single-center RCT [156] first reported that increased intensity (dose) (35 or 45 ml/kg/h) of CRRT was associated with lower mortality (42 or 43%) compared with a dose of 20 ml/kg/h (59%). Furthermore, in septic AKI patients, almost twice the survival rate was found in the larger dose group (45 ml/kg/h). However, these findings have more recently been challenged by two large recent multicenter RCTs.

These two recent key multicenter RCTs, the VA/NIH Acute Renal Failure Trial Network (ATN) [157] and the Randomized Evaluation of Normal versus Augmented Level of Renal Replacement (RENAL) [158] studies found that increased intensity (dose) of RRT was not associated with improved patient outcomes.

As given in Table 7, the ATN study used a strategy that allowed patients to switch between RRT modalities according to their hemodynamic status. RRT was provided as IHD in patients with hemodynamic stability and as either CRRT (mostly) or SLED (rarely) when hemodynamically unstable. No difference of 60-day mortality was found between less-intensity therapy arm and intensive arm (51.5 vs. 53.6%).

**Table 7:** Characters of ATN, RENAL and IVOIRE studies

	ATN	RENAL	IVOIRE
Design	Multicenter RCT	Multicenter RCT	Multicenter RCT
Country	USA	Australia and New Zealand	France, Belgium and Netherlands
Patients	AKI	AKI	AKI with septic shock
No. of patients	1124	1508	140
Modality	CVVHDF, SLED, IHD	CVVHDF	CVVH
Prescribed dose	CVVHDF: 21.5 versus 36.2 ml/kg/h SLED and IHD: 3 versus 6/wk	25 versus 40 ml/kg/h	35 versus 70 ml/kg/h
Delivered dose	CVVHDF: 22 versus 35.8 ml/kg/h SLED: 2.9 versus 6.2/wk IHD: 3 versus 5.4/wk	22 versus 33.4 ml/kg/h	33.2 versus 65.6 ml/kg/h
Mortality	60 days 51.5 versus 53.6%	90 days 44.7 versus 44.7%	90 days 50.7 versus 56.1%

*AKI* acute kidney injury, *CVVH* continuous venous–venous hemofiltration, *CVVHDF* continuous venous–venous hemodiafiltration, *SLED* sustained low-efficiency dialysis, *IHD* intermittent hemodialysis

In the second study (the RENAL study), 1508 patients were enrolled in 23 ICUs in Australia and New Zealand. All participants received CRRT, which were randomly assigned at an effluent flow of 25 or 40 ml/kg/h. The delivered dose was 22 and 33.4 ml/kg/h, respectively, and higher delivered/prescribed dose was found in less-intensity therapy (88 vs. 84%, *p* < 0.001). The primary outcome of 90-day mortality was 44.7% in both arms. In addition, both ATN and RENAL studies reported no difference in kidney recovery according to dialysis intensity (dose). However, hypophosphatemia was more common in the higher-intensity group.

These findings now strongly support the view that increasing dose intensity above 20–25 ml/kg/h does not deliver clinical benefits to critically ill patients with severe AKI and have established the current standard of care for “intensity (dose) of RRT” such patients.

### Intensity of RRT in septic AKI

Sepsis has been reported to account for approximately 50% of patients with AKI in ICU, and it has been hypothesized that modulation of pro-inflammatory cytokines in septic AKI might be beneficial [159]. Accordingly, a recent multicenter RCT focused on high-volume hemofiltration (HVHF) for septic AKI patients. The IVOIRE (hIgh VOlume in Intensive caRE) study [160] enrolled 140 AKI patients with septic shock from 18 ICUs in Europe and compared the efficacy of HVHF (70 ml/kg/h) with standard-volume hemofiltration (35 ml/kg/h). Although higher clearance of some solute (urea and creatinine) was reported in the HVHF group, there was no difference in 28-day or 90-day mortality between the two groups.

Two recent meta-analyses have further evaluated the issue of RRT intensity in AKI. Van Wert et al. [161] assessed 12 studies with 3999 patients, including 7 studies of CRRT, 3 of IHD, 1 of SLED and 1 of all three. These investigators found no benefit of more intensive RRT with regard to survival or dialysis dependence among survivors. A second meta-analysis [162] focused on HVHF (>50 ml/kg/h) for septic AKI patients and also found no difference in mortality between HVHF and standard-volume hemofiltration, but identified significantly higher rates of hypophosphatemia and hypokalemia in HVHF-treated patients.

As a consequence, the “Disease: Improving Global Outcomes (KDIGO)” AKI clinical practice guidelines [91] recommended that the “normal (standard) dose” of CRRT should in the range of 20–25 ml/kg/h and also recommended that, if IHD or SLED is chosen as the RRT modality for AKI, they should be set to deliver a Kt/V of 3.9 per week.

### Potential disadvantages of high intensity

There might be some potential complications which may counterbalance the advantage of higher clearance in high-intensity RRT. First, intensified therapy is reported to be associated with electrolyte disturbances such as hypophosphatemia or hypokalemia, which may do harm to renal, cellular, respiratory or cardiac function. Second, more attention should be focused on nutritional losses during CRRT. Increasing the intensity of RRT may double or triple the amount of amino acid or protein losses [163], as well as many micronutrient losses such as vitamins, selenium and folic acid [164]. Third, many antibiotics can be cleared significantly by RRT, and high-intensity RRT would make it more complicated to adjust the dose of antibiotics and could potentially generate periods of inadequate antibiotic levels, which, in turn, may impede the efficacy of antimicrobial therapy. Lastly, the RENAL study [158] found that high-intensity CRRT required more filters per day, indicating more clotting events and frequent interruption occurred during therapy. This effect could generate more costs, more manipulation and more red cell losses, which could also impact on patient well-being.

### Other aspects of dose

**Fig. 9:**
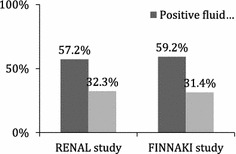
90-day mortality of RRT-treated AKI patients who were positive versus negative for the presence of fluid overload in RENAL and FINNAKI studies

The concept of the impact of dose on outcome has been explored only in terms of solute control. However, the term “dose” implies other aspects of RRT beyond solute control: volume control; timing of intervention; acid–base control; electrolyte control; and nutritional therapy optimization. Unfortunately, no RCTs have been conducted to test whether different modalities and/or techniques and/or intensity of RRT application can lead to different outcomes in relation to such more complex or extended aspects of “dose.” Despite such limitations, strong observational data [165, 166] have repeatedly found a clear correlation between a positive fluid balance and unfavorable outcome, suggesting that volume control may be an important to patient outcome as solute clearance (Fig. 9).

### Conclusions

There is no convincing evidence supporting the view that more intensive RRT can improve outcomes in AKI or septic AKI. At present, 20–25 ml/kg/h of effluent flow rate is recommended for CRRT practice, although sometimes effluent flow rates may need to be increased if there are frequent or prolonged interruptions of therapy. Kt/V remains the common available method of monitoring solute removal dose of IHD or SLED in AKI patients. However, adequacy of volume management and fluid balance should be also considered as an important “marker” for the intensity of therapy and requires targeted randomized controlled trials.

### Anticoagulation management in continuous RRT (CRRT)

Upon contact of blood with foreign material, blood cells and molecular pathways are triggered causing activation of coagulation and inflammation. Although biomaterials have improved, we still need anticoagulation to suppress this reaction and allow adequate hemodialysis or hemofiltration, especially during critical illness when coagulation is activated and natural anticoagulants are low.

Main anticoagulant measures include heparin and citrate. Additional non-anticoagulant measures such as lowering filtration fraction and pre-dilution, straight catheter course, avoidance of side holes and limiting blood-air contact are worthwhile [167].

*Heparin* Heparin inhibits thrombin formation by potentiating antithrombin and inhibiting factor XIIa. During critical illness, heparin has several drawbacks. Apart from circuit anticoagulation, heparin causes systemic anticoagulation and thereby increases bleeding risk. Furthermore, critical illness confers heparin resistance, because antithrombin may be low. Moreover, heparin binds to acute phase proteins and necrotic and apoptotic cells. Finally, heparin has pro-inflammatory effects by triggering the release of granular products from polymorph leukocytes and platelets [168].

Citrate. Citrate inhibits thrombin formation by decreasing ionized calcium (iCa), cofactor in the coagulation cascade. Because citrate is rapidly metabolized when entering the patient, citrate provides regional anticoagulation and does not increase bleeding risk. Citrate also inhibits the activation of granulocytes and platelets upon membrane contact and therefore increases biocompatibility.

*Citrate versus heparin* Randomized studies show that citrate is better tolerated, confers less bleeding, longer circuit life, a higher delivered dose and reduces the costs of CRRT [169].

*Citrate: anticoagulant, buffer and fuel* The use of citrate is, however, complex, because citrate is both anticoagulant and buffer. Anticoagulation depends on the chelation if calcium and thus on the citrate concentration in the filter blood. Anticoagulation starts when iCa falls below 0.5 mmol/l and is maximal below 0.25 mmol/l. In contrast, the buffer strength depends on the amount of unopposed strong anion entering in the patient’s circulation. The strong anion is most often sodium, but if acid citrate dextrose (ACD-A) is used, part of the anions are hydrogen, reducing buffer strength [170]. After metabolism of citrate, the unopposed sodium exerts the alkalizing effects by increasing the strong anion difference.

*Citrate*(C_6_H_5_O_7_) *confers energy* 0.59 kcal/mmol. One day of citrate CRRT provides 350–500 kcal, depending on the modality. However, ACD-A delivers up to 1000 kcal per day due to the associated glucose load. When lactate is used as buffer, about 550 kcal are delivered per day [170].

*Limitations* The safe use of citrate is limited by its metabolism in the mitochondria of liver, kidney and muscle. Mitochondrial metabolism is oxygen dependent. When citrate is not metabolized, it accumulates and lowers systemic iCa concentration. Total calcium (totCa) rises due to citrate chelation and calcium replacement. A rise in total to iCa ratio above 2.5 is the most specific warning sign of accumulation. Because citrate is not metabolized, SID increases less and metabolic acidosis can develop. Lactate concentration may be high, but not due to citrate accumulation but to underlying disease. Risk factors for accumulation are decompensated liver cirrhosis, severe systemic hypoperfusion and possibly sarcopenia: diseases associated with tissue hypoxia or loss of mitochondrial capacity. Although the incidence of citrate accumulation is low (1–3%) [171], its occurrence is associated with high mortality (up to 100%) due to underlying disease.

*Citrate and lactate* A lactate above 3.4 mmol/l was found to be predictive of citrate accumulation in patients with liver failure. However, adequate control of acidosis and no major electrolyte disturbances were observed despite a Ca ratio above 2.5 in 16% of the runs [172]. We questioned our database whether a lactate concentration above 4 mmol/l predicted citrate accumulation. We found that after 12 h, citrate accumulation (Ca ratio > 2.5) developed in 15/694 (2.2%) of the patients with lactate below 4 mmol/l and in 8/67 (10.9%) when lactate was above 4 mmol/l. Reason may be that during systemic inflammation hyperlactatemia is partially due to increased aerobic glycolysis, not to tissue hypoxia. Thus, a high lactate is not a contraindication for citrate anticoagulation per se and the risk of accumulation should be weighed against the risk of bleeding when using heparin or early clotting without anticoagulation. However, withholding citrate should be considered in case of a high lactate due to persisting systemic hypoperfusion or decompensated liver cirrhosis.

*Citrate monitoring* Monitoring of citrate includes iCa, acid base and totCa. Systemic iCa is used to guide calcium replacement. When both iCa and totCa decrease, calcium replacement should be increased. If iCa is low and the totCa/iCa ratio rises, citrate is accumulating. Whether we should stop citrate if Ca ratio is above 2.5 depends on metabolic control. If acid base is in equilibrium and iCa within range with additional replacement, citrate can be continued with close monitoring if risk of bleeding is increased.

The benefits and limitations of citrate are summarized in Table 8.

**Table 8:** Summary of the benefits and limitations of citrate anticoagulation

Benefits	Limitations	Monitoring
Compared to heparin Safety ↑ Tolerance ↑ Risk of bleeding ↓ Circuit life ↑ Delivered CRRT dose ↑ Biocompatibility ↑ Cheaper	Accumulation in case of persistent hypoperfusion and decreased mitochondrial capacity Difficult to understand Strict protocol is needed Adherence to the protocol is required	Systemic iCa and acid base balance 6–8 hourly Total calcium and total/iCa ratio once daily or more frequently if the risk of accumulation is high

*CRRT* continuous renal replacement therapy, *iCa* ionized calcium

### Conclusion

Citrate is first-choice anticoagulant in CRRT, but invest in understanding, stick to the protocol and monitor accumulation.

### SLEDD

The treatment of AKI in the ICUs knew some revolutions in the last twenty years, in particular in terms of techniques used, with the switch from nephrologists taking care to intensivist’s autonomy. IHD let place to CRRT, especially CVVH which is the most popular technique worldwide. But some hybrid techniques appeared, between CVVH and IHD, as sustained low-efficiency daily dialysis (SLEDD), that seem combined the advantages of the two older techniques [173].

In fact, there are very few studies and publications about SLEDD and its use remains confidential in the world. The technique seems to have some advantages in comparison with IHD as lower blood flow, slower fluid removal and solute clearance and less hemodynamic instability with a good solute control, the price is longer sessions (about 8–16 h). However, longer sessions imply increasing risk of circuit thrombosis and anticoagulation is often necessary at the same level as for CVVH.

Some studies have been completed in the last decade, but most of them were retrospective or observational studies with small sample size of patients and controversial results. A randomized controlled trial was published in 2012, with 115 patients included, comparing CVVH to SLEDD, showing that SLEDD was cheaper and reduced nursing time but without any clinical benefit [174]. Moreover, while SLEDD was done perfectly, CVVH treatment was inadequate with all the replacement fluid given in pre-dilution and with a filtration fraction about 29%. More recently, Zhang provided a meta-analysis that showed similar survival between CRRT and SLEDD and with a lower mortality in the SLEDD group when only observational studies are taking into account [175]. However, the sample size of observational studies is quite low (only 675 patients in 10 studies) and is potentially subject to allocation or selection bias. Moreover, a meta-analysis using only 7 RCTs was negative with in addition included a study from Egypt with only 80 patients but representing 30% weight in the analysis. [176]. Then, all these studies are underpowered and mostly observational and do not brought any signs of superiority for SLEDD in terms of outcome, solute control or metabolic equilibrium.

There are two possible benefits for SLEDD technique, the low cost and the time without therapy to mobilize the patient. In fact, financial benefit with SLEDD is really major when we use the specific machine with water treatment for dialysis solute, as the only cost is the price of the machine and the circuit/membrane consumables. But in that case you can only use the machine for SLEDD, when it is possible to use all the techniques (IHD, CVVH and plasmapheresis) with the new devices. Mobilization of patients is a major challenge in the modern ICUs, and intermittent techniques for RRT are superior in that case as we are able to treat the patients during the night and mobilize them during the day. But during the acute phase while mobilization is scarce and not priority this advantage is not crucial. The last but not least problem is with antibiotics, especially during the acute phase of sepsis, as the management of dosing is very difficult during RRT and is probably better to favor continuous methods than intermittent ones.

Finally, SLEDD is a hybrid technique between CVVH and IHD, with some advantages of the two methods but also disadvantages. The lack of strong studies and the relative low development of SLEDD in the world do not push physicians to use it. Clearly the main advantage of the technique is the low cost and the possibility of early mobilization of the patients. But the difficulty of antibiotics management, intermittent procedure that is not the best way for hemodynamic unstable patients and low level of middle size molecules clearance may limit its indication during the acute phase of AKI. The place of SLEDD might be during the chronic phase and should be explored in this way.

### How to assess recovery from AKI?

Whether or not the kidney recovers from an episode of AKI has important prognostic implications [177] and therefore deserves a correct assessment. At this moment, there is no consensus on the optimal timing to determine AKI recovery. For reasons of convenience, it is mostly assessed at hospital discharge, but others argue that the optimal timing would be 3 months after the start of AKI, because that is the earliest time-point that a formal diagnosis of CKD can be made [178].

Consensus on how to assess recovery is also lacking. The most commonly used parameters are: the absence of AKI criteria, the return to baseline creatinine or to within 1.1 or 1.25 times baseline, a 50% decrease from peak creatinine, a discharge creatinine that is less than 0.3 mg/dL above baseline, discharge CKD class or return to baseline eGFR. A recent analysis has demonstrated important differences in the proportion of complete recovery according to the severity of AKI and the definitions applied [179]. Some studies report renal recovery in survivors only, whereas others include the whole AKI population. It is evident that this will also affect the results. Whether the whole population or only survivors should be considered depends on the context. From a patient’s perspective and when the focus is on the need for nephrological follow-up, recovery in survivors is most meaningful. On the other hand, in an intervention trial on a therapeutic strategy to promote recovery, the whole population should be considered with mortality as a competing endpoint. While awaiting a consensus definition, it is important that studies on AKI recovery clearly describe their definitions, the population under evaluation, the severity of AKI and the proportion of CKD patients [179].

**Fig. 10:**
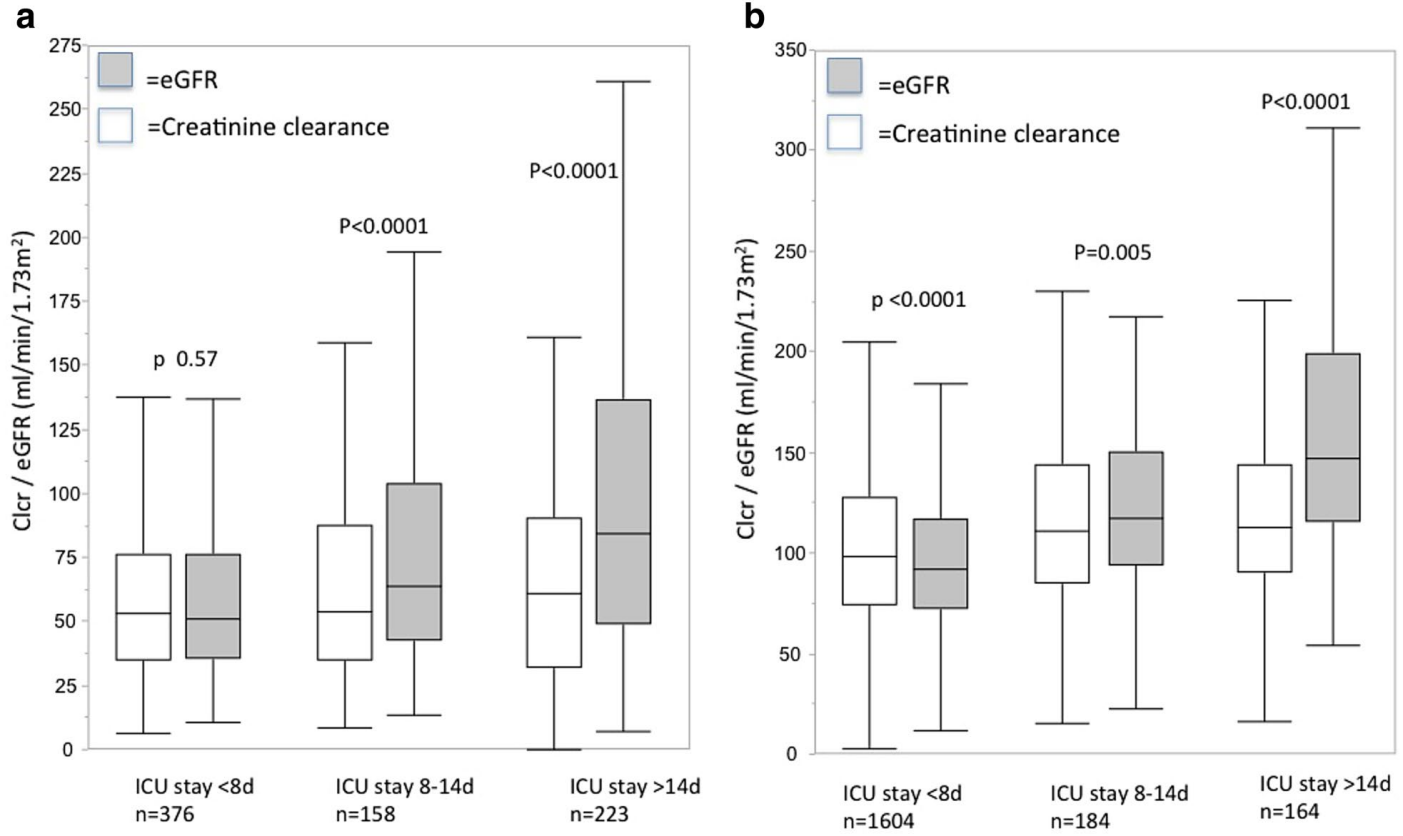
**a** eGFR (*gray boxplots*) and Clcr (*white boxplots*) (in ml/min/1.73 m^2^) at ICU discharge for subgroups of AKI patients with ICU stay <7 days, 7–14 days and >14 days. *Boxplots* show median and IQR, *whiskers* 10th and 90th percentile. **b** eGFR (*gray boxplots*) and Clcr (*white boxplots*) (in ml/min/1.73 m^2^) at ICU discharge for subgroups of non-AKI patients with ICU stay <7 days, 7–14 days and >14 days. *Boxplots* show median and IQR, *whiskers* 10th and 90th percentile (from [4]—with permission)

Another important drawback is that recovery in clinical settings is not evaluated with a gold-standard GFR measurement (e.g., insulin clearance) but with the only GFR parameters that are clinically available, i.e., either SCr or the derived eGFR that are compared with their baseline. This assessment assumes that the relationship between the true GFR on the one hand and creatinine and the derived eGFR on the other does not change during ICU stay. It is well known, however, that ICU patients, especially those with prolonged stay, develop muscle wasting with decreased creatinine generation. This may result in overestimation of recovery. The impact of this generally acknowledged phenomenon on the assessment of recovery has recently been quantified by comparing eGFR with the measured 24 h CCr at ICU discharge in AKI patients with different ICU stays. Whereas the two GFR measurements did not differ in patients with ICU stay below 7 days, eGFR became significantly higher than 24 h CCr in patients with ICU stay between 8 and 14 day, and this difference further increased in those with ICU stay above 14 days (Fig. 10a). As a result, assessing recovery with the 24 h CCr instead of the eGFR substantially reduced the incidence of complete recovery: In patients with ICU stay above 14 days it decreased from 60 to 30%. Measured creatinine excretion, as a parameter of muscle mass, decreased with increasing ICU stay and became significantly lower that the predicted creatinine generation (based on gender, age and weight) from an ICU stay above 7 days. Interestingly, the same results were seen in patients without AKI: here too the discharge eGFR was significantly higher than the 24 h CCr (Fig. 10b) and the creatinine excretion was significantly lower than the predicted generation in patients with longer ICU stay. In addition, the discharge creatinine in these long-stay patients was lower than baseline, again pointing to important muscle loss. The conclusion from this analysis is that comparing discharge eGFR or SCr with their baseline significantly overestimates recovery in patients with longer ICU stay. This also suggests that further increases of SCr after discharge do not necessarily point to further deterioration of kidney function but may simply reflect the restitution of muscle mass during revalidation [180].

Can we predict recovery? Reported risk factors for non-recovery are older age, pre-morbid GFR, comorbidity (e.g., diabetes), illness severity, cause and severity of AKI, fluid overload and modality of RRT [181]. More recent investigations have evaluated the accuracy of biomarkers for the prediction of (non)-recovery [182]. The available studies are small, and more research is needed.

In conclusion, timing and methods for assessing recovery from AKI significantly affect the results. The timing of recovery assessment should take into account muscle wasting during hospital stay. Consensus on when and how to determine recovery from AKI should be established.

## AKI: long-term outcomes

### Introduction

The strong and independent relationship between AKI and short-term mortality is well described in medical and surgical patients, in general ICU patients and in multitrauma patients [26]. Nearly half of patients with severe AKI, requiring RRT, die during hospital admission. In addition, major adverse long-term consequences in AKI survivors have been documented [177, 183–185]. The aim of this narrative review is to describe such long-term complications. We focus on AKI survivors’ renal recovery or lack thereof, the link between post-ICU renal morbidity and mortality and, finally, AKI survivors’ perception of their quality of life.

### AKI survivors’ long-term risk of CKD, ESRD and cardiovascular death

Depending on AKI severity and the presence or absence of pre-morbid CKD, approximately 2–30% of AKI survivors progress to ESRD and lifelong need for dialysis within 2–5 years after ICU discharge (Table 9) [177, 183, 184, 186–188]. Of 810 survivors to day 90 after severe AKI, requiring CRRT in the Randomized Evaluation of Normal versus Augmented Levels of RRT (POST-RENAL) study, 5.4% were dialysis dependent within a mean of 3.6 years [2]. CRRT intensity in ICU had no significant impact on the subsequent need for chronic dialysis. AKI severity, CKD and their relationship with non-renal recovery were further demonstrated in AKI survivors after major surgery [187]. Patients with severe postoperative AKI (of which 18.8% received acute dialysis) had an ESRD incidence of 5.1% (independent 22-fold increased risk compared to non-AKI patients) during a median follow-up of 4.8 years. In contrast, the corresponding incidence was 0.6% in mild AKI patients (doubled risk) and 0.3% in patients without AKI. In comparison, acute-on-CKD was independently associated with a 123-fold risk of progression to ESRD.

**Table 9:** AKI survivors and their long-term incidences and relative risks of mortality, CKD and ESRD

Authors [reference]	AKI severity	Study (n)	Follow-up^a^ (years)	Mortality		CKD		ESRD	
				%	Relative risk^b^ (95% CI)	%	Relative risk^b^ (95% CI)	%	Relative risk^b^ (95% CI)
Ishani et al. [6]	AKI only AKI + CKD	233,803	2.0^c^	54.3 64.3	2.48 (2.38–2.58) 3.24 (3.08–3.40)	NR	NR	2.5	13.0 (10.6–16.0) 41.2 (34.6–49.1)
Wu et al. [7]	AKI only Mild AKI Moderate AKI Severe AKI AKI + CKD	9425	4.8^c^	33.3 27.4 39.1 45.0 47.2	1.62 (1.45–1.81) 2.41 (2.11–2.75) 3.06 (2.66–3.53) 3.58 (2.91–4.41)	NR	NR	1.9 0.6 0.7 5.1 30.3	2.09 (0.97–4.52) 3.19 (1.27–8.03) 22.35 (11.9–42.1) 122.9 (66.8–253.9)
Pannu et al. [3]	AKI ± CKD AKI recovery AKI non-recovery	190,714	2.8^d^	30.8	1.0 1.26 (1.10, 1.43)	9.8	1.0 4.13 (3.38, 5.04)	2.1	NR
Gammelager et al. [8]	D-AKI ± CKD D-AKI only D-AKI + CKD	107,937	3.1^e^	NR	NR	NR	NR	3.8	6.2 (4.7–8.1)^c^ 11.9 (8.5–16.8) 2.8 (1.8–4.3)
Gallagher et al. [2]	D-AKI ± CKD	810	3.6^d^	31.9	NR	NR	NR	5.4	NR
Rimes-Stigare et al. [4]	AKI only	97,782	3.2^d^	21.8	1.15 (1.09–1.21)	6.5	7.6 (5.5–10.4)	2.2	22.5 (12.9–39.1)

*AKI* acute kidney injury, *CKD* chronic kidney disease, *ESRD* end-stage renal disease, *D-AKI* AKI requiring dialysis in ICU, *NR* not reported

^a^Mean or median follow-up

^b^Adjusted mortality rate ratio, incidence rate ratio, hazard ratio or odds ratio relative non-AKI patients or AKI recovery

^c^Considering survivors to hospital discharge

^d^Considering 90-day survivors

^e^Considering 180-day survivors

Even in patients with apparently normal baseline kidney function, the risk of progressive renal dysfunction is significant after AKI. In a Swedish cohort of almost 100,000 critically ill patients without pre-ICU kidney disease, approximately 5000 had AKI. Of these AKI patients, almost 3000 patients were still alive at day 90 [184]. During a median follow-up of 3.2 years, 21.8% of these patients died and 2.2% developed ESRD. In addition, 6.5% were diagnosed with de novo CKD (not requiring dialysis) in the national patient register during follow-up. Compared to non-AKI patients, these AKI survivors had an almost threefold increased mortality risk, a sevenfold increased risk of developing CKD and a 22-fold higher risk of developing ESRD. Similar incidences were observed in a Canadian cohort of survivors of hospital-acquired AKI assessed over a median of 2.8 years; 30.8% died and 2.1% developed ESRD [177]. Furthermore, in the same study, another 10% had a sustained a doubling of their SCr during follow-up. Finally, 42.1% of AKI survivors had albuminuria during follow-up in the POST-RENAL study suggesting persisting kidney damage in these patients [183].

CKD is a well-known risk factor for cardiovascular events and mortality [189], and both likely connect AKI with increased long-term mortality. In fact, patients with myocardial infarction (MI) complicated by AKI had higher risk of stroke, congestive heart failure, new myocardial infarction or death during 6 years of follow-up compared to patients with MI but without AKI [190].

The above-mentioned studies support the notion that AKI is a springboard to de novo CKD as well as to accelerated CKD progression, cardiovascular morbidity and mortality [190, 191]. Importantly, even transient AKI episodes resolving within 90 days after hospital discharge has been associated with a twofold increased risk of later CKD development [192]. Longitudinal surveillance of kidney function beyond 90 days therefore appears justified in AKI survivors.

### AKI survivors’ quality of life

Results from quality-of-life (QoL) assessments in critically ill patients with and without AKI are conflicting. Previous studies suggested poor long-term health-related QoL as a consequence of AKI in ICU [193, 194]. Reduced physical health compared to matched reference populations was mainly reported whereas self-reported mental health was less affected.

More recent studies suggest, however, that ICU patients’ QoL may be reduced already before ICU admission [195, 196]. Hofhuis et al. assessed pre-ICU QoL by proxy using Short-Form (SF)-36 and demonstrated significantly lower scores compared to an age-matched population [196]. In addition, after 6 months, survivors’ self-reported SF-36 score was significantly lower than their proxy-reported baseline score. No major difference between patients with and without AKI was found at the end of follow-up.

Similar results were found in the FINNAKI study [195]. The Euro Quality of life (EQ)-5D index was significantly lower on ICU admission as compared to an age- and sex-matched general population. Additionally, EQ-5D index was similar between AKI and non-AKI patients and, in contrast to the study by Hofhuis et al., did not change significantly during 6-month follow-up. In contrast, the perceived 6-month QoL (Visual Analogue Scale [VAS] component of EQ-5D) was similar to that of the reference cohort in all but the patients treated with RRT in ICU who had significantly reduced scores.

Health-related QoL in patients treated with RRT in ICU was assessed by using the SF-12 questionnaire in the POST-RENAL study [185]. Compared to the general population, the POST-RENAL patients had significantly lower physical and mental scores 3.5 years after ICU discharge. Importantly, the presence of albuminuria as an indicator of chronic kidney damage was independently associated with reductions in the physical component score of the SF-12.

### Conclusions

Severe AKI is a serious complication in critically ill patients and is associated with a high short-term mortality rate. Moreover, even mild and transient episodes of AKI appear to increase the long-term risks of chronic and end-stage renal disease and cardiovascular morbidity among survivors. Finally, evolution from acute to such chronic renal conditions likely explains the extremely high 5-year mortality seen in AKI patients as well as the reduced QoL in survivors after severe AKI. These findings suggest

### Control of host response with extracorporeal purification techniques

Sepsis remains the leading cause of death in ICUs nowadays, and the dream to find a technique to control host response is more alive than ever. Many techniques have been explored in this indication with good hopes after animal and preliminary studies but were disappointing after negative large randomized studies.

The idea to control sepsis start in the early nineties, with a study from Grootendorst, where pigs were hemofiltrated and effluent infused in healthy pigs, and they became sick and died only when effluent came from septic pigs and not when it came from healthy pigs. They proved for the first time that we were able to remove “bad humors” from septic animals and may control sepsis by this way [197]. From this point, many studies were conducted and blood purification techniques tested: HVHF, HCO membrane (HCO), plasmapheresis, adsorption and derived techniques (as coupled plasmafiltration and adsorption (CPFA), with variable results.

All these adjunctive treatments have the same objective; remove cytokines and other inflammatory or anti-inflammatory mediators to control the evolution of sepsis. Some theories have been developed to try to explain the potential interest of blood purification techniques. First the “peak concentration hypothesis” leaded by Ronco who hypothesized that the removal of deleterious peak of mediators secreted during sepsis by blood purification may control host response and avoid organ dysfunction. Then, Honoré thought that hemofiltration is also able to remove mediators from tissue in the “threshold modulation theory.” And last, Di Carlo with “the mediator delivery hypothesis” explained that mediators’ removal is more due to lymphatic wash-out by large volume of crystalloids infusion (which is the case during hemofiltration) than by removal in the effluent [198]. But unfortunately, our knowledge on immunology and sepsis is not sufficient to understand exactly what we have to remove and when to control host response in our patients.

In the last decade, important studies brought some answers about the efficacy of those techniques to decrease mortality in sepsis and to remove mediators. Only one large study with plasmapheresis has been completed, in Russia, a RCT with 106 septic shock patients included and a better survival for those treated by plasmapheresis. But this study is the lone study with these results and has not been reproduced [199]. Some RCTs were done with HCO membrane but not published yet and without positive results. A large multicenter RCT with HVHF tested (70 ml/kg/h) versus standard hemofiltration (35 ml/kg/h) on 140 septic shock patients included failed to show any benefit of HVHF [160] and confirmed the results of a monocenter Chinese RCT with 280 patients included comparing 50 ml/kg/h with 80 ml/kg/h. Also a large multicenter RCT in Italy with CPFA has been stopped prematurely for futility. More, a French RCT about hemofiltration started early (before AKI) in septic patients has been terminated prematurely for safety due to higher SOFA in the hemofiltration group in comparison with standard treatment [200]. Finally, only animal studies and small randomized or non-randomized human studies found positive results, when all the RCTs were negative but that one about plasmapheresis. One explanation is that we remove “bad things” with blood purification but also a lot of “good things” like antibiotics, vitamins or good mediators which is possibly more deleterious than host control is beneficial [200].

Currently, control of host response with blood purification techniques remains a dream. We continue to study new techniques, but with the results of last RCTs it is not reasonable to use them in routine practice or outside research protocols. Next studies should focus on reducing the deleterious impact of blood purification on antibiotics and vitamins removal to have a chance to reveal the possible benefit of immunomodulation in our septic patients.

### Extracorporeal epuration: beyond the kidney, the control of pathogen

By definition, the syndrome called sepsis implies a systemic inflammation, which changes the blood content with activated immune cells, release of mediators and hormones and presence of pathogen-related molecule (PAMPs) or tissue damage molecule (DAMPs). These modifications differ along time evolution of sepsis corresponding to modifications in blood constitution being responsible for waves of up- or down-regulation of inflammation [201]. The models using ex vivo or in vitro experiments with plasma from septic patients or animals have confirmed the presence of molecules in plasma that may alter cellular functions, even for healthy cells. As a consequence, “cleaning the blood or plasma” using extracorporeal circuit with different membranes or cartridges removing molecules by convection/adsorption/filtration is attractive for clinicians, despite the recent disappointing results of randomized clinical trials [200]. Recently, important proofs for an inflammatory mediation of septic-induced AKI have been reported, stimulating the development of cartridges targeting different plasma molecules [202] with the hope to control systemic inflammation. However, the targeted molecules to be removed remain elusive, since the knowledge of the positive versus negative components remains a challenge for clinician that is not clarified by the actual biomarkers, cell functions evaluation tests or tissue function tests. Although inflammation and kidney infiltration have been demonstrated to induce AKI, some infiltration might be protective for the tissues. As an example, the inflammatory monocyte (newly recruited) adhesion to renal vascular wall orchestrated by CX3CR1 activation has been shown to be protective for kidney injury in rat and human beings [203]. The I249 CX3CR1 allele is associated with both increased monocyte adhesiveness and reduced kidney damage in human septic shock. The strategy of care should focus on components well admitted to induce inflammation and/or tissue damage such as endotoxin or activated cells or both.

The plasma removal of endotoxin (EDTX) is seen as a hope to reduce inflammation during the acute phase of severe sepsis. The polymyxin B hemoperfusion to remove EDTX is used for several years in Japan as a routine therapy in septic shock. Before promoting this technique for a generalized use, randomized control trials have to prove the benefit for outcome parameters such as a reduced mortality, intensity of organ failure and length of stay. In this prospect, a first RCT has been reported in septic shock induced by an acute peritonitis in JAMA [204]. Despite the small number of enrolled patients (64) and the absence of EDTX plasma level measurements, this multicenter trial showed in a post hoc analysis a reduction in mortality rate at day 28 between control (53%) and treated groups (32%), with a decrease in vasopressor requirement. Recently, the prospective RCT performed on 232 septic shock patients related to peritonitis has been published [205]. Hemoperfusion with PMX membrane had no benefit on mortality at day 28 and day 90. Even after classification based on systemic inflammatory intensity (plasma IL-6 level), completion of 2 PMX sessions, after selection on surgical adequacy of surgical procedure, PMX hemoperfusion did not show any benefits on mortality. This RCT may have some limitations such as the relative modest number of patients and the absence of ET measurements. However, some positive aspects have to be also considered: the assessment of surgical procedure quality usually not quoted, the postoperative time delay to enroll the patients to reduce the anesthetic impact on hypotension, the protocolized cardiovascular resuscitation.

To conclude, even the concept of the control or removal of plasma mediators, especially EDTX remains attractive, no real proven benefit for outcome has been reported until now. The ongoing larger RCT in the USA (EUPHRATES) enrolling a larger number of septic shock patients with a high level of EDTX level will bring a definitive answer to the benefit of such a therapy.

## Liver support systems

### Introduction

The significant array of liver support systems available would suggest that the design, clinical utilization and end points for success remain poorly defined.

The literature is peppered with case series suggesting benefit and randomized controlled trials (RCT) that have failed to deliver mortality benefit.

Systems can be divided into (a) cleansing systems, (b) biological systems and (c) cellular transplantation.

The patient groups that these systems have been applied to are acute-on-chronic liver failure (AoCLF), alcoholic hepatitis, acute liver failure (ALF) and stable cirrhotics with profound pruritus.

The aims of any support system may be defined as biochemical improvement (bilirubin, direct and indirect and ammonia), clinical parameters (hemodynamic status, grade of hepatic encephalopathy (HE), coagulation parameters, pruritic scores) along with cytokines and immune function. In the patient with acute liver failure, there is a desire to stabilize and promote spontaneous liver regeneration and in the AOCLF, to stabilize and allow time for liver transplantation to be undertaken. There has been less examination of the role of such systems in hypoxic hepatitis or septic liver dysfunction.

### Cleansing systems

These may be considered standard renal replacement therapy (RRT), albumin dialysis systems, of which MARS is the most commonly reported where counter current to blood is run an albumin circuit within which there are adsorbent columns and renal filters. Plasma separation and adsorption are represented by Prometheus system where plasma is run over an adsorbent column and a renal dialysis filter prior to return to the patient. Plasma exchange is simple cleansing process exchanging the patient plasma with fresh-frozen plasma on a 1:1 ratio.

Biological systems. Single-pass albumin dialysis provides counter current slow dialysis (700 ml/min) of a solution of 5% albumin in a standard hemofiltration solution [206].

### Biological systems

These systems usually incorporate plasma separation; the plasma is then cleansed using an adsorbent column and is then run across hepatocytes (porcine or hepatoblastoma) and the treated plasma returned to the patient. The most commonly referenced systems are the bioartificial liver system (BAL) and extracorporeal liver assist device (ELAD) but others have also described and reported systems.

A more extreme form of biological liver support could be considered hepatocyte transplantation and auxiliary liver transplantation.

### Effects of therapies and end points

The effect of volume therapy has been reported to result in an 18% reduction in bilirubin [207], while by contrast increasing albumin levels increases bilirubin and decreases clearance when albumin dialysis is undertaken.

Acute kidney injury further contributes to impaired ammonia clearance in cirrhosis and institution of RRT decreases ammonia significantly [119] and rate of removal correlates with urea clearance.

Single-pass albumin dialysis (SPAD) studies have shown, in a rig, excellent clearance of ammonia, bile acids and bilirubin [206].

Studies with MARS have clearly shown improvement in biochemical parameters in several studies with falls in bilirubin, renal parameters and ammonia [208]. Equally frequently reported have been an improvement in hemodynamics and inflammatory cytokines. A RCT examining HE [208] in 70 patients demonstrated more rapid resolution of HE than in the control group but no mortality benefit.

Renal replacement therapy has also been shown to be very effective in decreasing ammonia [119]. Comparisons of MARS and Prometheus and SMT [209] showed that although Prometheus provided increased clearance of bile acids and bilirubin, MARS appeared to provide greater hemodynamic improvement. In a recent study, Sponholz [210] compared MARS and SPAD in a crossover design. Both systems were effective in decreasing bilirubin and bile acids while increasing albumin binding capacity. MARS was more effective at clearing urea and creatinine due to the relative flow rates, and the authors thus suggested RRT would be required in conjunction with SPAD.

Multiple studies of liver support, both biological and cleansing have shown benefit with regard to biochemical, physiological and mortality end points [211] have shown benefit in case series but unfortunately a similar pattern has not been seen in controlled trial [212, 213].

MARS compared with standard therapy in a large cohort of acute-on-chronic liver failure (AoCLF) in the RELIEF study showed improved biochemical parameters but no mortality benefit [214]. Kribben et al. reported the effects of plasma separation and adsorption in AoCLF [215]; overall therapy did not impact on survival although on subgroup analysis benefit was seen patients with a MELD > 30.

Various studies involving ELAD systems have been undertaken but have not shown mortality benefit.

The MARS system has also been studies in a multicenter study of ALF in France [216]. For all comers, there was no mortality benefit seen, although patients were transplanted at a median of 16 h post-randomization and listing. MARS therapy was associated with a greater chance of transplantation; while predictors of outcome were an acetaminophen etiology and lactate.

More recently, a study examining plasma exchange in ALF having progressed to grade 2 coma, with an etiology of largely acute and hyperacute patients showed a mortality benefit on intention to treat, with effect being largely observed in patients who did not proceed to transplantation [217].

### CO_2_ removal

ECCO2R (extracorporeal CO_2_ removal) systems are different from conventional extracorporeal membrane oxygenation (ECMO) systems in terms of blood flow, size and type of vascular access and anticoagulation requirements. Accordingly, such systems have little influence on the level of oxygenation, while they exert mainly a positive action by the mean of CO_2_ removal. Technological developments have led to modern venovenous minimally invasive ECCO2R systems proposed both for avoiding and shortening IMV in severe acute exacerbations (AE) of chronic obstructive pulmonary disease (COPD) patients and also for allowing ultra-protective ventilation in patients with ARDS. It is generally anticipated that technological developments could rapidly increase the number of suitable medical devices for minimally invasive ECCO2R. However, ECCO2R systems, even if much less invasive than ECMO systems, can carry their own complications, mainly in link with vascular access and with the anticoagulation regimen.

Stimulating results were reported in recently published pilot studies, both in the contexts of ARDS [218, 219] and of severe AE of COPD [220–223]. In a proof-of-concept study, Terragni demonstrated in 10 ARDS patients that ECCO2R permitted to safely reduce tidal volume from to 6.3 ml/kg (IBW) to 4.2 ml/kg [218]. The authors also reported beneficial effects on pro-inflammatory cytokines measurements and on morphometric pulmonary CT parameters. More recently, Bein reported the results of a RCT including 79 moderate-to-severe ARDS patients, aiming to establish a reduction in ventilator-free days [219]. The authors used of a pumpless arteriovenous CO_2_ removal system. Overall, the study was negative. However, the authors reported a reduction in mortality in the more severe patients, as defined by a PaO_2_/FiO_2_ ration less than 150 mmHg. They also reported less use of sedatives and a decrease in the IL-6 plasma level in the experimental group. However, they also reported three severe complications of the arterial cannulation.

In a multicenter retrospective study including 21 patients (14 COPD) with acute hypercapnic respiratory failure at high risk of NIV failure, Kluge et al. reported a low rate of intubation (10%) after initiation of a pumpless arteriovenous CO_2_ removal system [220]. However, they also reported a high incidence of bleedings related to the device (two major and seven minor) and one pseudo-aneurysm. As a consequence, the use of less invasive venovenous systems is now generally advocated, in order to diminish the rate of device-related complications. More recently, Burki et al. reported the results of a pilot open study using a venovenous system ensuring ECCO_2_R of about 80 ml/min (which is close to 1/3 of the average value of the physiological CO_2_ elimination in normal adults at rest) at blood flow rates comprised between 350 and 500 ml/min [221]. The corresponding vascular access was achieved by mean of a specific double lumen 15.5 F central venous catheter. In a group of patients at high risk of NIV failure, the authors reported that intubation could be avoided for all seven patients. Using another device, Del Sorbo et al. reported the results of a matched cohort study with historical control including in the experimental group 25 COPD patients with severe AE at high risk of NIV failure [222]. Risk of being intubated was three times lower (*p* = 0.047) than in the control group, with intubation rates of 12 and 33%, respectively. Hospital mortality was significantly lower in the experimental group, but with large 95% CI: 8% (95% CI 1.0–26.0) versus 33% (95% CI 18.0–57.5). All the three previously referenced study confirmed the ability of these different devices to effectively reduce PaCO_2_ values and to increase pH values [220–222]. Finally, some data are also available with regard to the interest of ECCO_2_R in already intubated COPD patients, either as a primary therapeutic option or after NIV failure. In five such severe patients treated shortly after intubation, Abrams et al. reported a median delay of 4 h (with a maximal duration of 21.5 h) between ECCO_2_R initiation and extubation [223]. They also reported a mean duration of ECCO_2_R of 193.0 + 76.5 h as well as a mean time to ambulation after ECCO_2_R initiation of 29.4 + 12.6 h However, Burki et al. reported less positive results in a subgroup of COPD patients placed on ECCO_2_R much later after intubation [221].

To date, no vast RCT has evaluated the efficacy of minimally invasive ECCO_2_R either in ARDS or in patients experiencing severe hypercapnic AE of COPD. Such trials are now urgently warranted. Specifically for AE of COPD (an acute-on-chronic condition), it will be of great importance to assess by such RCTs the efficacy and safety of the method on relevant clinical endpoints, not only limited to short-term ICU events. If positive, such studies could also greatly improve patient-centered outcomes, not only limited to the short-term ICU course. It also could help to ethically implement such expansive technologies in the hospital’s practice.

### Cell cycle arrest biomarkers: new weapons for a new battle

AKI is becoming an important health concern not only because the syndrome is a deadly condition in itself, but also because it represents a gateway to CKD [224]. AKI is a syndrome with high mortality due to comorbidities and management challenges, especially in the critically ill patients [225]. AKI, however, is more than that. Even minimal kidney damage due to an insult (exposure) in the tubular or glomerular structure may evolve into progressive apoptosis and fibrosis and possibly a devastating glomerular destruction with inevitable hyper-filtration of the remnant parenchyma. Thus, AKI is a near and present danger that has ramifications for the rest of a patient’s life. Several efforts have been made in recent years to standardize the definition/classification of AKI and, above all, to make an early diagnosis of acute kidney damage/dysfunction. This effort has included the discovery and validation of new biomarkers of AKI.

In spite of a growing body of publications, many new biomarkers have not yet transitioned to clinical routine because of a series of unresolved issues [226]. The first is the lack of specificity for AKI of some molecules. The amount of false-positive cases associated with elevation of the biomarkers caused by acute and chronic comorbidities in patients without AKI has often been too high. The second is lack of sensitivity of some markers, particularly at the earliest stages of kidney injury. A third is the absence of clinically relevant and validated cutoff values that help guide use of the biomarkers to trigger appropriate interventions and changes to patient management. In addition, a major concern has been that once significant damage has occurred, the possibility to modify the clinical course and especially the recovery phase was considered minimal or absent. The extent to which this may or may not be true is unknown, but a significant number of patients with AKI are known to recover kidney function [227]. Therefore, the general consensus is that at least some kidney tissue can be salvaged and earlier detection and intervention are likely to benefit the patient. This may be especially true at the earliest stages of stress and injury when it may be possible to prevent further damage and preserve remaining renal capacity, for example, by removing potentially injurious exposures such as nephrotoxic drugs and by providing extra supportive measures such as heightened attention to fluid and hemodynamic management [91].

There is unanimous agreement that a specific plan should be undertaken to fight AKI and its consequences. A strategic move of the scientific community to prevent, protect, diagnose and cure AKI is definitely needed not only to save many lives from the acute disorder, but also to avoid the evolution into CKD either by reducing the level of injury or by facilitating healing and recovery of the damaged parenchyma. However, all these approaches have been hindered by the lack of reliable methods for early diagnosis of the injury and an early identification of the patient at risk.

Recently, the US Food and Drug Administration made an important step forward in the battle against AKI and its consequences. The FDA cleared the marketing of the NephroCheck Test (Astute Medical Inc. San Diego, USA), a rapid test for the quantitative measurement of the cell cycle arrest biomarkers tissue inhibitor of metalloproteinase-2 (TIMP2) and insulin-like growth factor binding protein-7 (IGFBP7) [228]. The combination of the two biomarkers ([TIMP2]·[IGFBP7]) measured by the test seems to be highly predictive of which patients will develop moderate-to-severe AKI in the next 12–24 h.

Early work in the international multicenter Sapphire study of 728 critically ill patients showed that elevation of the combination of biomarkers measured by the NephroCheck Test is specific to AKI (i.e., is not caused by other comorbidities such as sepsis or CKD) and provides a strong signal or “renal alarm” to identify when a patient is at imminent risk of developing AKI [229]. These urinary biomarkers are believed to be elevated in response to renal tubule cell stress or early injury associated with the types of exposures known to cause AKI. A primary clinical cutoff value (0.3) for the combination of the two biomarkers was derived from the Sapphire study data and verified in a new cohort of 153 critically ill patients (Opal study) [230]. This cutoff was selected to have high sensitivity for the primary endpoint of moderate-to-severe AKI in the next 12 h, with the intent to be used in routine clinical practice to identify patients at high risk of AKI who therefore are candidates for kidney-sparing management strategies such as those outlined in the KDIGO guideline for high-risk patients [91]. A second, high specificity cutoff (2.0) was selected and verified to identify the subgroup of patients who are at the highest risk of AKI and who therefore might be appropriate for more active interventions. Both cutoffs (0.3 and 2.0) were subsequently validated in a 23 site study of 408 critically ill patients in the USA (Topaz study) using clinical adjudication to determine the primary endpoint of moderate–severe AKI [231].

The NephroCheck quantitatively measures the combination of the two cell cycle arrest biomarkers ([TIMP2]·[IGFBP7]) both by point of care techniques and other laboratory platforms, thus expanding the availability of the test worldwide [232].

According to the recent publication of the Acute Dialysis Quality initiative consensus group [233], there is a need for early identification of damage or risk of AKI, especially in those patients in which creatinine is still negative but biomarkers are positive. In this sense, NephroCheck may be used alone, or in combination with other biomarkers of AKI as a discriminating test to alert physicians. All these considerations assume that putting the diagnostic clock ahead by 12–24 h compared to the clinical clock can make a difference. We are particularly convinced that this is the case. Early diagnoses or assessment of risk of injury may not only contribute to the identification of the cause of AKI and hopefully mitigate its effects, but also may help to identify patients in which, due to high susceptibility, even a small exposure may cause a severe injury. Even a subclinical (creatinine negative) injury, which may appear to be negligible, can produce a significant parenchymal damage [22]. This may be underestimated due to the presence of a significant RFRin the kidney and the absence of clinical signs and symptoms [23]. The injury, however, reduces the functioning renal mass and produces a progressive increase in kidney frailty with a remarkable susceptibility to future injuries. This process may be the gateway to CKD.

We must, therefore, use all the tools we have to raise the level of patient care and escalate the battle against AKI. A reliable, validated and widely available test with a specific cutoff threshold has been requested by clinicians for a long time. A simple urinary biomarker test to screen critically ill patients for the risk of AKI is something that is likely to be a useful new weapon in the battle against AKI. In this area, FDA has taken an important step to provide us with a new tool that is an early alert of which patients are at imminent risk. We should take the next step in using this new tool to help us improve the care of our patients.

## Abbreviations

ACD-A: acid citrate dextrose
ADHF: acute decompensated heart failure
AE: exacerbations
AKI: acute kidney injury
AKIN: Acute Kidney Injury Network
ANA: antinuclear antibody
ANCA: anti-neutrophil cytoplasmic antibodies
ANP: atrial natriuretic peptide
AoCLF: acute-on-chronic liver failure
AoCRF: acute-on-chronic kidney disease
AP: alkaline phosphatase
ARDS: acute respiratory distress syndrome
ARF: acute renal failure
ATN: acute tubular necrosis
BAL: bio-artificial liver system
biAP: bovine-derived intestinal
APBSE: bovine spongiform encephalopathy
CAN: contrast-associated nephropathy
CARS: cardio-abdominal renal syndrome
CCr: creatinine clearance
CEUS: contrast-enhanced ultrasound
CHI3L1: chitinase-3-like protein 1
CKD: chronic kidney disease
COPD: chronic obstructive pulmonary disease
CPFA: coupled plasmafiltration and adsorption
CRRT: continuous RRT
CVC: central venous catheters
CVVH: continuous venovenous hemofiltration
CVVHD: venovenous hemodialysis
DAMPs: tissue damage molecule
DNA: deoxyribonucleic acid
ECCO2R: extracorporeal CO_2_ removal
ECMO: conventional extracorporeal membrane oxygenation
EDTA: ethylenediaminetetraacetic acid
EDTX: endotoxin
eGFR: estimated GFR
ELAD: extracorporeal liver assist device
ESRD: end-stage renal disease
FENa: fractional excretion of Na^+^
GFR: glomerular filtration rate
GN: glomerulonephritis
HCO: high cutoff
HE: hepatic encephalopathy
HRF: delineating hepatorenal failure
HUS: hemolytic uremic syndrome
HVHF: high-volume hemofiltration
IAH: intra-abdominal hypertension
IAP: intra-abdominal pressure
iCa: ionized calcium
IGFBP7: insulin-like growth factor binding protein-7
IHD: intermittent hemodialysis
kDa: Kilodaltons
KDIGO: Kidney Disease: Improving Global Outcomes
KIM-1: kidney injury molecule-1
LDH: lactate dehydrogenase
LPS: lipopolysaccharide
MAP: mean arterial pressure
MCP: membrane cofactor of proteolysis
MDRD: modification of diet in renal disease
MI: myocardial infarction
NAC: *N*-acetylcysteine
NGAL: Neutrophil gelatinase-associated lipocalin
NSAIDS: nonsteroidal anti-inflammatory drugs
PAMPs: presence of pathogen-related molecule
PCR: polymerase chain reaction
PD: peritoneal dialysis
QoL: quality of life
RAAS: renin–angiotensin–aldosterone system
RCT: randomised controlled trial
recAP: recombinant AP
RFR: renal functional reserve
RIFLE: Risk Injury Failure Loss End-Stage Kidney Disease
RPGN: rapidly progressive GN
RPP: renal perfusion pressure
RRT: renal replacement therapy
RRTs: renal replacement therapies
SBP: spontaneous bacterial peritonitis
SCr: serum creatinine
SF: Short-Form
SLED: sustained low-efficiency dialysis
SLEDD: sustained low-efficiency daily dialysis
SPAD: single-pass albumin dialysis
TIMP2 IGFBP3: tissue inhibitor of metalloproteinase 2 and insulin-like growth factor binding protein 3
TIMP2: tissue inhibitor of metalloproteinase-2
TIMP-2*IGFBP7: tissue inhibitor of metalloproteinase 2 and insulin-like growth factor binding protein 7
totCa: total calcium
TTP: thrombotic thrombocytopenic purpura
UO: urine output
VAS: visual analogue scale
